# The Neural Representation of Prospective Choice during Spatial Planning and Decisions

**DOI:** 10.1371/journal.pbio.1002588

**Published:** 2017-01-12

**Authors:** Raphael Kaplan, John King, Raphael Koster, William D. Penny, Neil Burgess, Karl J. Friston

**Affiliations:** 1 Wellcome Trust Centre for Neuroimaging, UCL Institute of Neurology, University College London, London, United Kingdom; 2 UCL Institute of Cognitive Neuroscience, University College London, London, United Kingdom; 3 Clinical, Education and Health Psychology, University College London, London, United Kingdom; 4 UCL Institute of Neurology, University College London, London, United Kingdom; Oxford University, UNITED KINGDOM

## Abstract

We are remarkably adept at inferring the consequences of our actions, yet the neuronal mechanisms that allow us to plan a sequence of novel choices remain unclear. We used functional magnetic resonance imaging (fMRI) to investigate how the human brain plans the shortest path to a goal in novel mazes with one (shallow maze) or two (deep maze) choice points. We observed two distinct anterior prefrontal responses to demanding choices at the second choice point: one in rostrodorsal medial prefrontal cortex (rd-mPFC)/superior frontal gyrus (SFG) that was also sensitive to (deactivated by) demanding initial choices and another in lateral frontopolar cortex (lFPC), which was only engaged by demanding choices at the second choice point. Furthermore, we identified hippocampal responses during planning that correlated with subsequent choice accuracy and response time, particularly in mazes affording sequential choices. Psychophysiological interaction (PPI) analyses showed that coupling between the hippocampus and rd-mPFC increases during sequential (deep versus shallow) planning and is higher before correct versus incorrect choices. In short, using a naturalistic spatial planning paradigm, we reveal how the human brain represents sequential choices during planning without extensive training. Our data highlight a network centred on the cortical midline and hippocampus that allows us to make prospective choices while maintaining initial choices during planning in novel environments.

## Abstract 2

Using neuroimaging and computational modelling, this study explains how the human brain represents initial versus subsequent choices during spatial planning in novel environments.

## Author Summary

We are remarkably adept at inferring the consequences of our actions, even in novel situations. However, the neuronal mechanisms that allow us to plan a sequence of novel choices remain a mystery. One hypothesis is that anterior prefrontal brain regions can jump ahead from an initial decision to evaluate subsequent choices. Here, we examine how the brain represents initial versus subsequent choices of varying difficulty during spatial planning in novel environments. Specifically, participants visually searched for the shortest path to a goal in pictures of novel mazes that contained one or two path junctions. We monitored the participants’ brain activity during the task with functional magnetic resonance imaging (fMRI). We observed, in the anterior prefrontal brain, two distinct responses to demanding choices at the second junction: one in the rostrodorsal medial prefrontal cortex (rd-mPFC), which also signalled less demanding initial choices, and another one in the lateral frontopolar cortex (lFPC), which was only engaged by demanding choices at the second junction. Notably, interactions of the rd-mPFC with the hippocampus, a region associated with memory, increased when planning required extensive deliberation and particularly when planning led to accurate choices. Our findings show how humans can rapidly formulate a plan in novel environments. More broadly, these data uncover potential neural mechanisms underlying how we make inferences about states beyond a current subjective state.

## Introduction

Goal-directed behaviour rests on being able to rapidly evaluate the potential consequences of future actions. For example, consider the neuronal processing required for planning a new route home when a road you normally take is closed. Although previous studies have implicated anterior prefrontal regions in planning [1–5], it has been difficult to tease apart the relative contributions of different prefrontal cortex (PFC) regions (i.e., rostral versus caudal or lateral versus medial PFC) that respond to choices later in a sequence [6–7]. Moreover, the neural representation of how we rapidly make a series of novel choices remains unclear, because planning studies generally rely on extensive learning about the outcomes of alternative choices [2–5,7].

Here, we ascertained whether different anterior PFC regions signal uncertainty about novel sequential choices in a distinct manner during plan formation. Specifically, we were interested whether rostrodorsal medial PFC (rd-mPFC), a brain region associated with imagining/simulating potential choices [8–10], might be biased towards responding to choices later in a sequence, even in the absence of prior learning about the consequences of choices.

**Fig 1:**
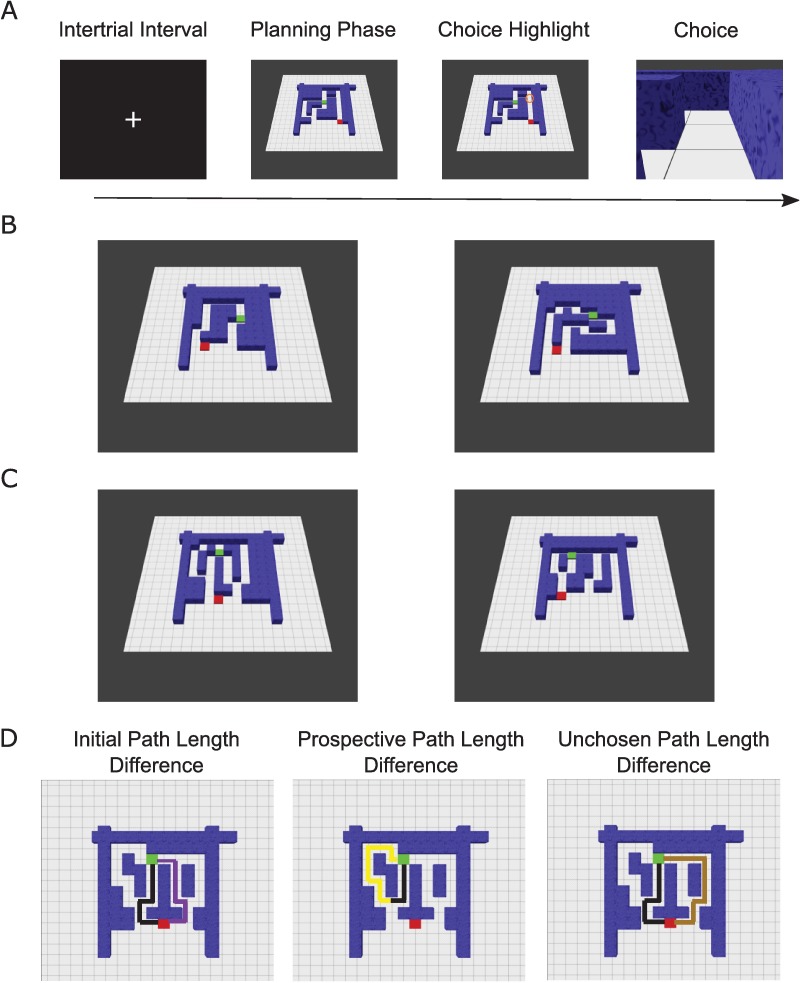
Task. (A) During a 3.25-s planning phase, participants had to infer the shortest path from the starting point in maze (a red square) to the goal location (green square) and remember the chosen direction for each choice point along the shortest path. Half of the mazes (shallow mazes) had two paths and only one choice point (red square), whereas the other half (deep mazes) had four paths and two choice points (red square and another point further in the maze). After 3.25 s, a choice point was highlighted (choice highlight) for 250 ms. The highlighted location could either be the red square or the second choice point along the shortest path for deep mazes. In shallow mazes, only the red starting location was highlighted. Crucially, for deep mazes, participants were tested on one choice point before starting the next trial. Subsequently, the choice period featured a first-person viewpoint of the highlighted location, where participants had a maximum of 1.5 s (Deep Maze mean: ~545 ms; Shallow Maze mean: ~440 ms) to choose the correct direction on the shortest path (left, forward, right, or equal) with a button box. Immediately following the button press, an intertrial interval (ITI) screen appeared for 1.5 s before a new trial began. (B) Left: Example shallow maze trial with a large path length difference (less demanding choice). Right: Example shallow maze trial with a small path length difference (demanding choice). (C) Left: Example deep maze trial with a small path length difference (demanding) initial choice at the red square and large (easy) path length difference at the second (prospective) choice point. Right: Example deep maze trial with a large (easy) path length difference initial choice at the red square and small (demanding) path length difference at the second (prospective) choice point. Deep mazes contained a combination of small, medium, or large path length differences at first (initial) and second (prospective) choice points. (D) Overhead view (not shown during the experiment) of three example mazes showing which path lengths contribute to each parametric regressor in our fMRI analyses: initial (left), prospective (centre), and unchosen path length differences in deep mazes. Initial path length differences in shallow mazes represent the difference between the only two available paths. The black line highlights shortest path.

We created a spatial planning task that would require little to no learning in which participants could call on an internal model of space deployed during exploration of the physical world [11]. Our task required participants to choose the shortest route between a start and goal location during functional magnetic resonance imaging (fMRI) scanning: participants viewed one of 220 mazes with either two routes (shallow mazes) or four routes (deep mazes) to the goal. Shallow mazes only had one choice point at the start location, whereas deep mazes also offered a second choice point deeper into the maze. This design enabled us to see how responses to plan formation were modified by the depth of prospection (i.e., the number of choice points) and the uncertainty about those choices (i.e., the difference in lengths between the two available paths from each choice point). After planning their route, participants were asked to make a decision—at a specified choice point in a given maze—about the direction of the shortest path (i.e., optimal choice) to the goal location. This gave us an additional measure (reaction time [RT]) to quantify the uncertainty about a choice beyond the difference in available path lengths (Fig 1A). As with shallow mazes, participants were only prompted to make one choice after seeing a deep maze, but until the choice point was highlighted, they did not know which choice point (starting point or the choice point further in the maze) would be probed.

## Results

### Behaviour

Participants made correct choices 84.0% of the time (standard deviation [SD] = 5.13%; *n* = 29) during the fMRI experiment, with an average RT of 492 ms (SD = 150 ms). In deep mazes, when participants were prompted with choices that were at junctions deeper in the maze (i.e., the second/prospective choice point of a two choice sequence), they made correct choices 84.9% (SD = 9.89%) of the time, with an average RT of 671 ms (SD = 172 ms). There was no significant difference (t(28) = 1.84; *p* = 0.077; SD = 6.62%) in behavioural accuracy (percentage of correct choices) between deep (mean = 85.2%; SD = 6.33%) and shallow trials (mean = 82.9%; SD = 5.88%). In contrast, there was a significant difference in RT (t(28) = 14.3; *p* < 0.001; SD = 39.3 ms), with greater RTs for deep (mean = 545 ms; SD = 148 ms) versus shallow trials (mean = 440 ms; SD = 152 ms). Notably, mean RTs were not correlated with accuracy across participants (r = 0.257; *p* = 0.178).

**Fig 2:**
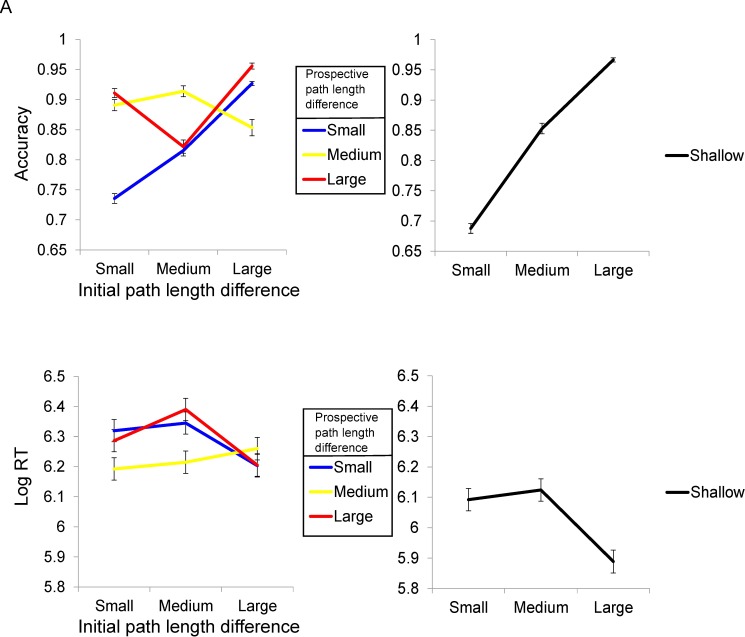
Behavioural Results. (A) Accuracy during choice phase. Left: Significant interaction (*p* < 0.001) for initial versus prospective path length differences in deep mazes. Deep mazes are split by small–small, small–medium, small–large, medium–small, medium–medium, medium–large, large–small, large–medium, and large–large path length differences at the initial choice point (i.e., the shortest options for either choice at the starting location) and the two available paths at the prospective choice point, respectively. Right: Significant positive linear trend in accuracy (*p* < 0.001) with increasing path length differences for shallow mazes. Shallow mazes are split by small, medium, and large path length differences. (B) Log RT during the choice phase. Left: Significant interaction (*p* < 0.001) for initial versus prospective path length differences in deep mazes. Deep mazes are split by small–small, small–medium, small–large, medium–small, medium–medium, medium–large, large–small, large–medium, and large–large path length differences at the initial choice point (i.e., the shortest options for either choice at the starting location) and the two available paths at the prospective choice point, respectively. Right: Significant negative trend (*p* < 0.001) in log RT with increasing path length differences in shallow mazes. Shallow mazes are split by small, medium, and large path length differences. See S1 Data for participant data.

Investigating the effect of path length differences on participant choice accuracy and RT in deep mazes, we observed a significant interaction between initial (i.e., first choice point) and prospective (i.e., at the second choice point) path length differences for both accuracy (F(2,27): 25.6; *p* < 0.001; Fig 2A) and RT (F(2,27): 11.4; *p* < 0.001; Fig 2B). There was a significant positive linear trend for accuracy and initial path length differences (F: 19.4; *p* < 0.001) but no similar linear trend for RT (F: 0.18; *p* = 0.674). Notably, we observed positive and negative significant linear trends with prospective path length differences for accuracy (F: 13.9; *p* = 0.001) and RT (F: 7.5; *p* = 0.011), respectively.

In shallow mazes, we observed a significant main effect of path length difference for both accuracy (F(2,27): 173.1; *p* < 0.001; Fig 2A) and RT (F(2,27): 52; *p* < 0.001; Fig 2B). As expected, there was a significant positive linear trend for accuracy (F: 354.6; *p* < 0.001) with larger path length differences, whereas there was a significant negative trend with RT (F: 81; *p* < 0.001; Fig 2). In deep mazes, accuracy was much lower when there were both small initial and prospective path length differences (Fig 2A).

We then investigated the influence of path length differences at the initial choice point on prospective choice behaviour. Unsurprisingly, when participants were prompted with the prospective choice point, we observed a significant (*p* < 0.05) main effect of the prospective choice path length difference (F(2,27): 6.57; *p* = 0.005; S1 Fig) on these choices and a linear increase in accuracy with larger path length differences (F(2,27): 8.47; *p* = 0.007). However, we found no significant difference in prospective choice accuracy when split by the initial path length difference (F(2,27) = 0.887; *p* = 0.424; S1 Fig). Investigating prospective choice RT, we observed a main effect of prospective choice RT based on the path length difference of the prospective choice point (F(2,27) = 6.40; *p* = 0.005; S1 Fig) and also when split by the (unprobed) initial path length difference (F(2,27) = 5.70; *p* = 0.009; S1 Fig). Similar to choice performance, there was a negative linear trend for higher prospective choice point RT with smaller path length differences at the prospective choice point (F(2,27) = 6.0; *p* = 0.021). However, we did not observe a significant linear decrease in RT when we split prospective choice RTs by the path length difference of the initial choice/starting point (F(2,27) = 3.1; *p* = 0.089). Taken together, these results suggest that the path length difference of the initial choice did not affect performance on prospective choices but did influence deliberation time (i.e., RT).

### fMRI Analysis

To assess the impact of planning sequential choices with varying processing demands, we classified deep maze trials by the path length difference between the shortest path and the other paths separately (i.e., initial, prospective, and unchosen path length differences; see Fig 1D for schematic of each path length comparison and S4 Table for list of regressors). Additionally, we asked whether subsequent choice behaviour (RT and accuracy) as well as other aspects of the planning task (e.g., the length of the shortest available path and whether the first or second choice was prompted during deep maze trials) also explained brain activity during the planning phase. To summarize, we included the following parametric modulators for deep maze trials: the path length difference between the two shortest paths present at the starting point (Initial Path Length Difference), the path length difference at the optimal second choice point (Prospective Path Length Difference), the path length difference between the longest/least viable path in the initially unchosen direction and the shortest path (Unchosen Path Length Difference), participants’ subsequent log RT during the choice phase (Log RT), the length of the shortest available path, whether participants answered the subsequent choice trial correctly or not (Accuracy), and whether participants were prompted to make an initial or prospective choice (Prompted Choice). Importantly, the same parametric modulators were included for shallow maze trials except for Prospective Path Length Difference, Unchosen Path Length Difference, and Prompted Choice.

We only report clusters that survive family-wise error (FWE) correction for multiple comparisons (*p* < 0.05) at the statistical threshold of *p* < 0.005 uncorrected. The only exception is in the hippocampus, where all reported activations contain a peak-voxel that survives (*p* < 0.05) small-volume correction (SVC) for the bilateral hippocampus.

### Prospective Path Length Difference

**Fig 3:**
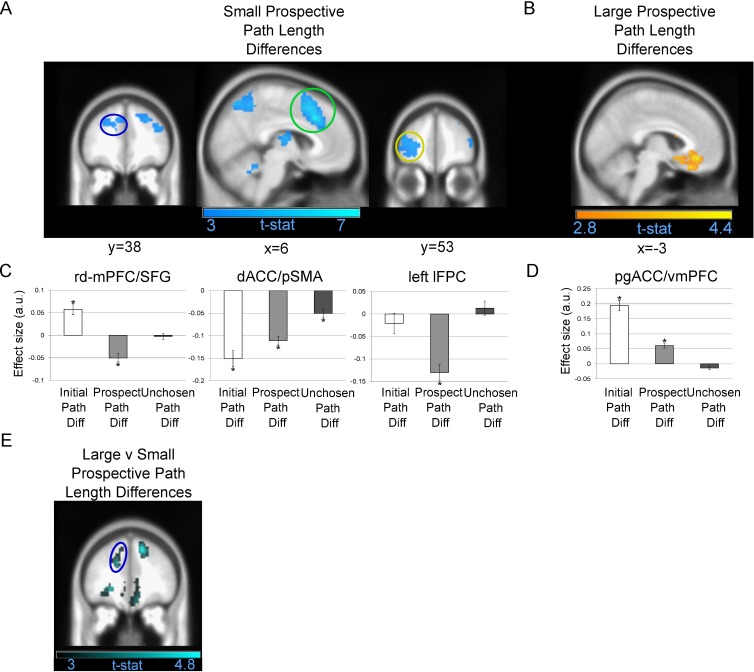
Prefrontal responses to prospective path length differences. (A) Regions significantly responding to smaller prospective path length differences. Left: Coronal image showing rd-mPFC/superior frontal gyrus (SFG). Centre: Sagittal image showing dACC/pSMA. Right: Coronal image showing left lFPC. (B) Pregenual anterior cingulate cortex/ventromedial PFC (pgACC/vmPFC) region significantly engaged by larger prospective path length differences. (C) Effect size for an 8-mm sphere around the rd-mPFC/SFG (left), dACC/pSMA (centre), and left lFPC (right) peak voxels that responded to smaller prospective path length differences displayed in A for three parametric modulators: initial (including both deep and shallow mazes), prospective, and unchosen path length differences (mean ± standard error of the mean [SEM]). (D) Effect size for an 8-mm sphere around the pgACC/vmPFC peak voxel that responded to larger prospective path length differences. For both C and D, asterisks indicate a significant correlation (*p* < 0.05) with path length differences. A positive effect size represents a BOLD correlation with larger path length differences, whereas a negative effect size represents a correlation with smaller path length differences. (E) Images centred on rd-mFPC peak in A, which was the only region featured in a that significantly responded to decreasing prospective versus initial choice path length differences in shallow mazes. All highlighted regions survived cluster-level FWE correction at *p* < 0.05 and are displayed at an uncorrected statistical threshold of *p* < 0.005. Corresponding coordinate from the Montreal Neurological Institute (MNI) template image listed below each brain image. See S2 Data for individual effect size data.

We first asked whether, during deep maze trials, there were fMRI responses specifically related to inferences about the prospective choice point, i.e., blood-oxygen-level dependent (BOLD) changes related to choosing between the two paths at the second choice point that were not fully explained by path length differences at the initial choice point. We observed a very large cluster peaking in dorsal anterior cingulate cortex/pre-supplementary motor area (dACC/pSMA; x = 6; y = 23; z = 37; Z-score: 5.08; Fig 3) with a sub-peak extending into rd-mPFC; x = −15; y = 38; z = 34; Z-score: 2.8; Fig 3) that responded to smaller prospective path length differences. Notably, there were also significant clusters in lateral frontopolar cortex (lFPC; x = −27; y = 53; z = 4; Z-score: 3.86; Fig 3), posterior parietal cortex (PPC; x = 3; y = −73; z = 55; Z-score: 4.41), left inferior temporal cortex (x = −57; y = −43; z = −17; Z-score: 3.68), and right cerebellum (x = 30; y = −55; z = −26; Z-score: 3.73; S8 Table).

Given that the rd-mPFC activation was a small sub-peak in a very large cluster centred on dACC/pSMA, we wanted to confirm whether there was truly a robust rd-mPFC signal selectively related to planning prospective choices and whether this signal differed from the other prefrontal responses observed in dACC/pSMA and lFPC. We therefore conducted a paired *t* test comparing responses to prospective path length differences versus initial path length differences in shallow mazes. We observed a significant rd-mPFC sub-peak (x = −15; y = 38; z = 28; Z-score: 3.61; Fig 3D) that responded to smaller prospective versus initial path length differences. The cluster covered the peak rd-mPFC voxel from the previous contrast and was centred on left dorsolateral prefrontal cortex (dlPFC; x = −18; y = 17; z = 43; Z-score: 4.33; see S2 Fig for images of dlPFC peak). Crucially, this cluster was much smaller than the previous rd-mPFC result and did not include the dACC/pSMA region that responded to prospective path length differences. Likewise, we observed no significant difference in lFPC responses to smaller prospective versus initial path length differences. Of particular interest, the significant effect in rd-mPFC was driven by its significant response to both large initial and smaller prospective path length differences (Fig 3C)—a pattern that was not observed in lFPC or dACC/pSMA.

The null result suggesting that lFPC does not respond to smaller prospective versus initial path length differences in shallow mazes should be interpreted with caution. Our general linear model (GLM) based on path length differences did not distinguish whether these fMRI results were due to the number of paths or the depth of planning. Indeed, when using a Shannon entropy model that compared RT-fitted uncertainty for prospective path length differences versus the absolute value of the difference between all four available paths lengths (see S1 Text for details), we found that both rd-mPFC and lFPC selectively responded to prospective uncertainty (S1 Text).

In the reverse contrast, larger path length differences at the prospective choice point elicited responses in pregenual anterior cingulate cortex/ventromedial PFC (pgACC/vmPFC; x = −3; y = 38; z = −11; Z-score: 3.69; Fig 3B). Notably, this finding is in contrast to a model-based analysis (see Supplemental Results, S1 Text) in which no parallel activation in pgACC/vmPFC related to decreasing prospective uncertainty was observed. This is possibly due to the inclusion of all path length differences and not just the two shortest paths available at either choice point.

### Initial Path Length Difference

We also examined whether in both deep and shallow planning trials there were regions that responded to the difference between the two shortest path lengths available at the initial/first choice point (See Fig 1D for illustration). We found that larger path length differences at the initial choice point elicited responses in the temporoparietal junction (TPJ)/angular gyrus, vmPFC (S3 Fig), and posterior cingulate cortex (PCC; see S3 Fig and S7 Table). Notably, rd-mPFC (t(28): 2.41; *p* = 0.023) but not lFPC (t(28): −0.468; *p* = 0.644; Fig 3C) significantly responded to increasing initial path length differences (see Table 1 for rd-mPFC and lFPC *t*-values related to other parametric regressor of interest). It is important to note that this vmPFC cluster only responding to large initial path length differences (S3 Fig) is rostral and superior to the pgACC/vmPFC cluster responding to both large initial and prospective path length differences.

**Table 1:** rd-mPFC and lFPC responses to different parametric regressors.

**rd-mPFC/SFG responses**	**t-statistic (df = 28)**
Subsequent Log RT	2.59*
Length of Shortest Path	−2.88*
Performance	−0.35
**lFPC responses**	**t-statistic (df = 28)**
Subsequent Log RT	3.63*
Length of Shortest Path	−3.00*
Performance	0.11

Asterisks signify *p* < .05; *t* > 2.05.

Abbreviations: df, degrees of freedom.

Following our results related to larger path length differences, smaller path length differences at the first choice point elicited responses in the dACC/pSMA, along with right dlPFC, anterior insula, and PPC (see S7 Table). The dissociation between regional responses increasing and decreasing with initial path length differences reflects similar responses to larger versus smaller reward prediction errors observed during value-guided choice [12–14].

### Unchosen Path Length Difference

**Fig 4:**
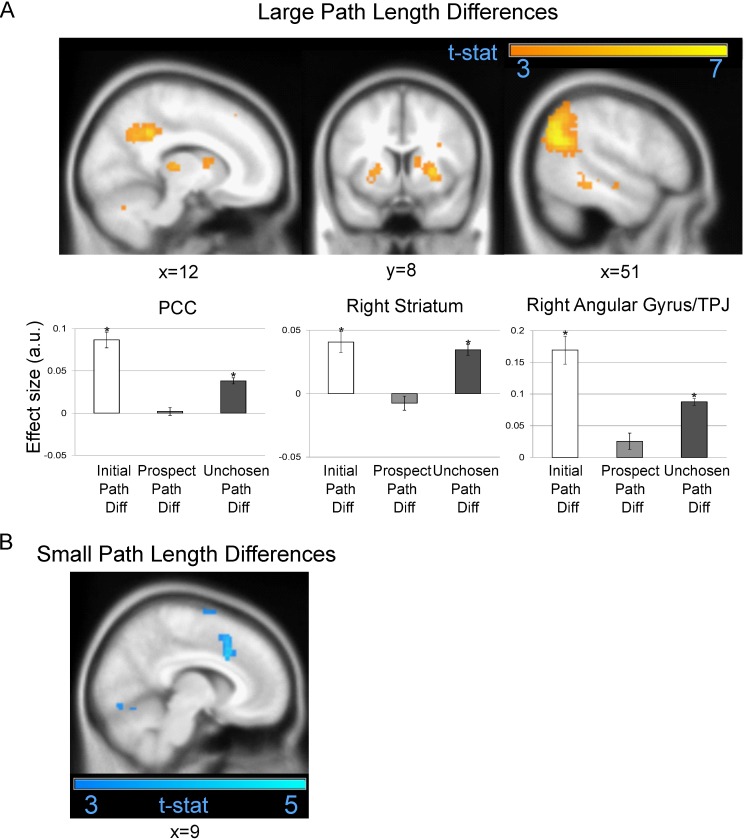
fMRI activations related to unchosen path length difference. (A) Regions that significantly responded to larger unchosen path length differences between the shortest and unchosen path. Top left: Sagittal image showing posterior PCC. Top centre: Coronal image showing right striatum. Top right: Sagittal image showing right angular gyrus. Bottom: Effect size for an 8-mm sphere around the PCC (left), right striatum (centre), and right angular gyrus (right) regions that responded to larger unchosen length differences displayed in A, for three parametric modulators: initial (deep and shallow mazes), prospective, and unchosen path length differences (mean ± SEM). Asterisks indicate a significant correlation (*p* < 0.05) with path length differences. A positive effect size represents a positive BOLD correlation with larger path length differences, whereas a negative effect size represents a correlation with smaller path length differences. (B) dACC region significantly responding to smaller unchosen path length differences. All highlighted regions survived cluster-level FWE correction at *p* < 0.05 and are displayed at an uncorrected statistical threshold of *p* < 0.005. See S3 Data for individual effect sizes.

In a separate comparison, we examined responses to the difference between the shortest and the least viable counterfactual/unchosen path (i.e., what regions corresponded to an exhaustive search or pruning of all potential paths). We found that larger unchosen path length differences engaged the right angular gyrus/TPJ (x = 51; y = −61; z = 25; Z-score: 5.98; Fig 4A and S9 Table), which was the strongest response we observed in any region relating to a path length difference regressor. Additionally, we found PCC (x = 12; y = −46; z = 37; Z-score: 4.93; Fig 4A) and right striatum (x = 27; y = 8; z = 1; Z-score: 4.58; Fig 4A) responses related to larger unchosen path length differences. Notably, in our Shannon Entropy model analysis, which did not have a specific parametric regressor accounting for unchosen path length differences, we found that right angular gyrus/TPJ and PCC both significantly related to increasing prospective uncertainty (Supplemental Results, S1 Text). Taken together, these analyses suggest that angular gyrus and PCC prune unviable paths in deep mazes that afford demanding prospective choices.

In contrast, smaller unchosen path length differences engaged dACC (x = 9; y = 17; z = 34; Z-score: 4.41; Fig 4B) and bilateral lateral occipital cortex (LOC; left: x = −24; y = −88; z = −4; Z-score: 4.71; right: x = 27; y = −91; z = 10; Z-score: 5.1; S9 Table). Our post hoc region of interest (ROI) analyses revealed that neither rd-mPFC (t(28): −0.19; *p* = 0.85) nor lFPC (t(28): 0.399; *p* = 0.693) significantly encoded the unchosen path, further suggesting that these regions corresponded to rapid sequential inference but not necessarily an exhaustive search of all possible paths.

### Subsequent RT

**Fig 5:**
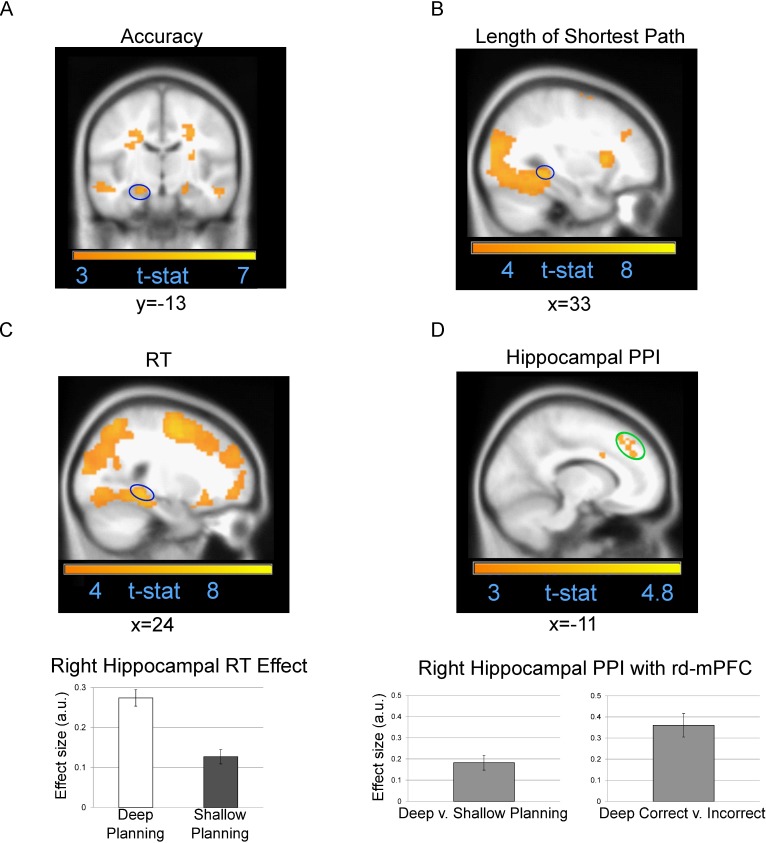
Hippocampal contributions to planning. (A) Coronal image showing higher left hippocampal activity (circled in blue) during planning prior to correct versus incorrect choices. Subthreshold right hippocampal activity that was higher for correct choices is also visible. (B) Sagittal image showing ventral temporal activity extending into right posterior hippocampus (circled in blue) that positively correlated with the distance of the shortest route between the starting and goal location. (C) Top: Sagittal image showing posterior right hippocampal activity during planning that positively correlated with subsequent log RT. Bottom: Effect size for an 8-mm sphere around right posterior hippocampus peak voxel showing that the correlation with log RT is significantly higher (*p* < 0.05) for deep versus shallow planning trials. All hippocampal peak voxels presented survive correction for multiple comparisons (*p* < 0.05) across the whole hippocampal volume, but clusters are shown at *p* < 0.005 uncorrected for visualization purposes. (D) Top: Sagittal image showing medial extent of rd-mPFC (peak voxel same as 3A) that exhibited increased functional connectivity with the right posterior hippocampus in deep versus shallow maze planning trials. Bottom left: Effect size for an 8-mm sphere around rd-mPFC peak voxel (mean ± SEM) showing significantly increased functional connectivity with hippocampus for deep versus shallow planning trials. Bottom right: Hippocampal rd-mPFC functional connectivity (mean ± SEM) was significantly higher during deep planning trials prior to correct versus incorrect choices. See S4 Data for individual effect sizes presented in 5C and 5D.

Asking whether other aspects of mazes (beyond path length differences) influenced neural responses during planning, we investigated whether any fMRI signals during planning correlated with subsequent RT during the choice phase. During planning, fMRI signals in an extremely large portion of cortex—peaking in visual cortex—positively correlated with subsequent RT (S10 Table). The large visual cortical cluster also encompassed ventral temporal regions extending into the bilateral posterior hippocampus (left: x = −27; y = −37; z = −11; Z-score: 4.97; small-volume corrected (SVC) *p* < 0.001), peaking in the right hippocampus (x = 24; y = −37; z = −8; Z-score: 5.0; SVC *p* < 0.001; Fig 5). Notably, the right posterior hippocampus peak showed a significantly stronger relationship with subsequent RT in deep versus shallow maze trials (t(28) = 2.71; *p* = 0.011; Fig 5C).

We also observed similar significant responses in smaller clusters in middle temporal gyrus and dACC (see S10 Table). Likewise, we observed significant (*p* < 0.05) positive correlations with increased subsequent RT in right angular gyrus/TPJ (t(28) = 3.53; *p* = 0.002) and PCC (t(28) = 3.01; *p* = 0.006) regions relating to larger unchosen path length differences, which provides additional evidence that these regions prune unviable paths during deep planning trials. The only negative correlation with subsequent RT was in the insula extending into a large portion of white matter (x = 27; y = −10; z = 10; Z-score: 4.72).

### Length of Shortest Path

We investigated which regions responded to the distance of the shortest available path length (i.e., whether the optimal path was distal or proximal to the goal location, irrespective of the other available paths). We observed responses in inferior occipital cortex extending into right posterior hippocampus (x = 33; y = −37; z = −8; Z-score; 4.79; SVC *p* < 0.001; Fig 5B) that correlated with increasing length of the shortest available path to the goal, along with dACC (S11 Table). Conversely, bilateral TPJ, pgACC/vmPFC, rd-mPFC, precuneous, posterior superior temporal sulcus, and lateral PFC (see S4 Fig and S11 Table) correlated with decreasing distance of the shortest available path length.

### Accuracy

Further characterizing the functional contribution of different brain regions, we asked if the responses of different regions during the planning phase related to whether participants subsequently made a correct or incorrect choice. We observed a left hippocampal activation (x = −18; y = −13; z = −17; Z-score: 3.87; SVC *p* = 0.044; Fig 5A) that preceded correct choices with a subthreshold activation in right anterior hippocampus. Additionally, bilateral cerebellum and motor cortex activations during the planning phase related to correct choices (S12 Table). However, the spatial extent of these performance results should be interpreted with caution, because the hippocampal cluster extended into a large portion of white matter.

Conversely, there was a significant dACC/pSMA cluster (x = 6; y = 17; z = 49; Z-score: 6.89; S5 Fig) that preceded subsequently incorrect choices, which was the strongest activation observed in any contrast. We then tried to determine whether this response was feedback related, because it could have been due to an unobserved choice point. However, we found no significant difference between deep and shallow planning (t(28) = 1.32; *p* = 0.198; S5 Fig). Likewise, adding a regressor encoding whether the initial or prospective choice point was highlighted in deep mazes (Prompted Choice) did not modify the robustness of the dACC/pSMA activation. Notably, we also observed significant clusters in bilateral anterior insula and intraparietal sulcus (IPS) preceding incorrect choices (S12 Table).

### Deep versus Shallow Planning Effects

Investigating whether any regions responded differently to initial path length differences in deep versus shallow mazes, we found that a large cluster in PPC responded more strongly to smaller initial path length differences in shallow versus deep mazes. Likewise, we also observed smaller but significant clusters in premotor cortex (PMC) and dlPFC (S6 Fig and S13 Table). We did not observe any other significant clusters responding to initial path length differences in deep versus shallow mazes.

Next, we investigated whether during the planning phase there were any regions outside of the hippocampus whose responses correlated with subsequent RT for deep versus shallow planning trials differently. We found that visual cortex and right PMC correlated with increasing RT more strongly during shallow planning trials (S6 Fig and S13 Table) but did not find any other significant responses. When splitting responses to the length of the shortest path, we observed that inferior temporal cortex and superior frontal gyrus (SFG) responded to longer optimal path lengths more during deep versus shallow planning trials. Lastly, left LOC, left PPC, and right IPS responses to incorrect choices were higher for shallow planning trials (see S6 Fig and S13 Table).

### Psychophysiological Interactions of the Hippocampus

We conducted a psychophysiological interaction (PPI) [15] analysis of whether the right posterior hippocampal region (Fig 5C) relating to longer subsequent RT was coupled with rd-mPFC as a function of planning depth (mazes affording single versus sequential choices). We tested which regions exhibited increased coupling with hippocampus for deep versus shallow maze planning trials. Taking an 8-mm sphere around the rd-mPFC peak that selectively responded to smaller prospective path length differences (x = −15; y = 38; z = 34), we observed significantly increased coupling between the hippocampus and rd-mPFC for deep versus shallow planning (t(28) = 2.69; *p* = 0.012; Fig 5D). Notably, the hippocampus coupled more strongly with rd-mPFC than any brain region (peak voxel, x = 12; y = 47; z = 28; Z-score: 3.95; in a separate cluster that did not survive FWE cluster correction *p* < 0.05 at the whole-brain level). We did not observe any other significant clusters that coupled with the hippocampus anywhere else in the brain for deep versus shallow planning.

To assess the functional relevance of hippocampal coupling with rd-mPFC during deep planning, we conducted a separate GLM analysis splitting deep planning trials based on whether the subsequent choice trial was answered correctly or not (see Supplemental Methods in S1 Text for details of the GLM). We found that hippocampal coupling with rd-mPFC was significantly higher for correct versus incorrect deep planning trials (t(28) = 3.04; *p* = 0.005; Fig 5D).

## Discussion

Using fMRI and a novel spatial planning paradigm, we examined how different brain regions respond to prospective versus initial choices. We observed two prefrontal regions, lFPC and rd-mPFC, that responded to smaller prospective path length differences (i.e., demanding second-step choices) during planning. Distinguishing the role of these two regions in planning prospective choices, we found that rd-mPFC most strongly responded to deep mazes with larger path length differences at initial/starting choice points and smaller path length differences at prospective choice points (Fig 4). In contrast, lFPC responded to smaller path length differences at prospective choice points without any significant response related to initial path length differences. Notably, we observed hippocampal signals that correlated with subsequent choice accuracy and response time, particularly in mazes affording sequential choices. Additionally, we observed enhanced hippocampal functional connectivity with rd-mPFC during deep maze planning that was higher prior to correct choices. In parallel, we found PCC and angular gyrus responses relating to unchosen paths during sequential planning, whereas vmPFC/pgACC activity related to larger initial and prospective path length differences. In what follows, we relate our prospective spatial planning findings to the wider decision-making literature and to the hippocampal and parietal signals we observed. We then speculate on potential planning computations that might occur during our task.

### The Role of rd-mPFC versus lFPC during Planning

Highlighting distinguishable contributions to prospective planning for medial versus lateral anterior prefrontal regions, we find that rd-mPFC responds to difficult prospective choices while maintaining easier initial choices, whereas lFPC responds to prospective path length differences without being significantly modulated by initial path length differences. These findings are in line with the perceived capacity of anterior PFC to exploit recent reward trends during value-guided choice [16] and spatial navigation [2]. More specifically, our findings suggest that rd-mPFC might be guiding computations related to chaining the whole sequence of choices, whereas lFPC more exclusively relates to robust planning at the second, more prospective choice point independent of the initial choice. Alternatively, when there are increased computational demands at the initial choice point, rd-mPFC might deactivate when it is not clear what the first step should be, allowing lFPC or dACC to take over more robust prospective planning. The ability of lFPC and dACC to help robustly compute second-step choices is in line with previous findings related to counterfactual signals in FPC [6,17–18] and dACC signals related to strategic shifts in decision-making [19–20], along with the smaller unchosen path length dACC signals presented here. Notably, our behavioural results showed initial choice path length differences modulate subsequent RT during prospective choices but not whether the choice was correct or not, which suggests more than one underlying computation occurring related to prospective planning. Taken together with our anterior PFC findings, these data broadly implicate at least two distinct anterior prefrontal computations when planning next-step choices in novel environments—one rapid and another more deliberative computation related to prospective planning.

This lateral versus medial distinction parallels previous research on anterior PFC, where lateral areas are believed to process stimulus-independent (i.e., counterfactual) information, whereas medial areas are engaged by stimulus-oriented information [21]. Furthermore, prospective choices responses in rostral mPFC were primarily dorsal, but the exact location of responses was highly variable over participants, which may relate to the high anatomical variability between individuals in an evolutionarily complex region [1]. Still, our observation of prospective planning responses throughout rostral mPFC is consistent with recent findings showing that different populations in mPFC contribute to internal strategy shifts (see [22–24] for human evidence and [25–28] for rodent evidence) and persistent activity in order to reevaluate sequential choices [29].

Our result showing increased lateral FPC responses to prospective path length differences might relate to the perceived function of FPC as a simultaneous evaluator of multiple options, perhaps due to a higher sampling capacity (i.e., capable of maintaining more information) than rd-mPFC. Simultaneous evaluation of multiple options is necessary whether a decision is a sequential choice problem or not and is supported by the putative role of FPC in the rapid learning of novel abstract rules [30] and counterfactual choice [6,17,31]. Further work could focus on the influence of working memory load or cognitive control on types of planning [32–34] and how or whether different cognitive demands determine how a plan is formed or implemented and which prefrontal structures (e.g., dACC versus lFPC or rd-mPFC) are engaged.

### Hippocampal Responses during Spatial Planning

Decisions often rely on prospection during multi-step events in order to anticipate a potential outcome, which is a process commonly linked with hippocampal-based memory ([7,35–37]; see [38] for review). Furthermore, spatial planning in novel environments is usually associated with the use of a hippocampal-based internal model formed by exploration of the physical world [11], yet corresponding evidence of hippocampal involvement during on the fly planning without extensive prior learning has been lacking. Here, we present evidence of posterior hippocampal responses related to increased deliberation for novel sequential choices and anterior hippocampal responses that relate to choice accuracy. Although our experiment is more akin to a perceptual decision-making task than virtual navigation, our results are still consistent with the role of the hippocampus during navigational planning [5,39–41]. More specifically, posterior hippocampal activity related to increasing distance between the start and goal locations—along with higher right posterior hippocampal activity prior to longer choice RT in deep mazes—helps link our spatial decision-making results to the putative role of the right posterior hippocampus, which is thought to encode memory related to the spatial layout of an environment [42–44].

In novel environments, posterior hippocampal functional connectivity with rd-mPFC increased during deliberative planning for deep mazes and was highest before choice trials that were answered correctly. Likewise, a recent fMRI study has shown increased anterior prefrontal coupling with the hippocampus during remembering and planning upcoming trajectories to goal locations [5]. Oscillatory coupling between the posterior medial temporal lobe and rostrodorsal portions of mPFC has been observed during dynamic spatial imagery [45], and our data add further support that coupling between these regions could relate to comparison of novel choices with previous experience [38].

Notably, the hippocampus is also thought to play a key role in rapid incidental learning [46–47]. Our anterior hippocampus activation related to spatial planning performance illustrates how the hippocampus can contribute to quick model-based inferences during tasks with little to no learning. Yet it is still unclear how one-shot episodic learning might contribute to hierarchical planning. Investigating the neural representations of novel decisions might help uncover contextualization processes important for decision-making (e.g., chaining together sequential choices as a single decision outcome) and episodic memory (e.g., chaining together individual learned representations into a cohesive episode).

### Potential Computations Underlying Plan Formation and Implementation

We have elaborated on the distributed neural responses that relate to rapid prospective planning, but the precise computations required for our task are unclear. One disadvantage of our task is the inability to probe the time scale of plan formation and implementation in novel environments, particularly when choice accuracy and RT are influenced differently by path length differences. Most planning studies test after extensive training and are biased towards action-by-action evaluation without the need to maintain prior choices [3–4,48–50]. With extensively trained choices, the neural computations leading to increased decision implementation/RT are well studied [51–52]. On the other hand, the anterior prefrontal regions selectively responding to prospective uncertainty make evaluations that are more akin to rapid approximation of the best looking trajectory or jumping ahead to the most important sub-goal, which are neural computations that have not been as well explored. Interestingly, this “jumping ahead” process resembles computations that facilitate generalization between similar sequential states (successor representations) during episodic learning [53–55] and also best-first forward search models [56]. Exploring the interactions between the successor representation, time scales, and heuristic pruning during plan formation could potentially help disclose the computations underlying rapid and efficient multi-step planning in novel environments [57–59].

### The Role of vmPFC and dACC during Spatial Planning

Given that our experiment does not separate responses related to plan formation and implementation, the role of the vmPFC and dACC in our task is unclear. We observed dACC/pSMA responses related to an exhaustive comparison of path lengths (comparing the shortest path with every other available path), with additional responses related to increased deliberation, longer distance between starting and goal locations, and, most prominently, subsequently incorrect choices. Taking into account the importance of the dACC in model updating [60–61], it is not surprising that dACC responses would relate to uncertainty about potential trajectories at different choice points. However, due to the poor temporal resolution of our task, it is unclear whether dACC/pSMA responses are related to checking back on an uncertain initial choice point [62], focusing on one choice point for an extended period of time [63], performance monitoring [64], or increased cognitive control caused by difficult choices (see [61, 65] for an in-depth discussion of the potential role of dACC in these behaviours).

In contrast with dACC, vmPFC responses did not relate to comparisons of all available path lengths. Although subgenual portions of vmPFC responded to larger path length differences at both initial and prospective choice points, we did not observe any vmPFC signals that correlated with subsequently correct choices or quicker subsequent RT. A potential explanation for this result could be that vmPFC initially helps locate task-relevant sub-goals and signals an update of the current state [19,66].

Our findings also uncovered parietal responses that parallel activations observed in dACC and vmPFC. Smaller path length differences at both initial and prospective choice points engaged structures like PPC that have previously been implicated in value-guided decision-making when there is surprise and/or time pressure [60,67]. Notably, in other areas of the parietal lobe, right angular gyrus/TPJ and PCC responses during planning related to large initial and unchosen path length differences but also correlated with increased subsequent choice RT. One way to reconcile these seemingly contradictory results is that angular gyrus and PCC might be responding to irrelevant paths that need to be pruned/ignored [68], which could then help us suddenly proceed/shift [69–71] to a subsequent decision during planning. Planning studies informed by recent work investigating divisive normalization during multi-alternative choice [72] and dACC–PCC interactions when pursuing unlikely choices [20] can potentially isolate the biophysical mechanism underlying pruning irrelevant alternatives during sequential decision-making.

Notably, vmPFC, TPJ, and PCC responses to larger initial path length differences (i.e., certainty) overlap with a brain network commonly observed during value-guided choice [14,73]. Specifically, regions that increased with the precision of beliefs about choices overlap with regions that respond to reward differentials, i.e., greater value differences between chosen and unchosen options during value-guided decision-making [12–13,74]. Likewise, PPC and dACC/pSMA responses are also observed both during difficult value-guided choices (i.e., smaller value differences between chosen and unchosen options) [12,14,75] and smaller initial path length differences. This suggests a similar mechanism guiding probabilistic choice in both spatial and value-guided decision-making, regardless of whether an explicit reward, like food or monetary gain, is present.

### Internal World Models and Prospective Choice

We observed increased coupling between the hippocampus and rd-mPFC during sequential plan formation that also predicted subsequent performance. Notably, resting-state fluctuations in these same regions—along with angular gyrus and PCC—are also correlated and form the default network [76–78]. Promising clues relating internal models of the physical world to resting default network fluctuations might come from investigating hippocampal sharp-wave ripples: spontaneous oscillations that co-occur with the reactivation (and pre-activation) of hippocampal place cell ensembles [79–83]. Indeed, a recent study in macaques has shown that ripples selectively influence ongoing activity in the default network but not other resting-state networks [84]. Additionally, reactivation of hippocampal representations of previously learned goal locations has been observed during pre-navigational planning in familiar environments in humans [5]. Despite these promising findings, further research is still necessary to determine whether endogenous hippocampal interactions with cortical midline regions reflect reactivation/exploration of internal states in order to prepare decision-making networks for upcoming novel choices [59,70,85–86].

### Conclusion

We present a task adapted from rodent spatial navigation that enabled us to elucidate core neural computations underlying our ability to make fast and robust multi-step inferences in the absence of prior learning [85–87]. Our findings highlight a unique contribution of brain regions that do not respond to an exhaustive search of possible options during planning like caudal PFC and premotor regions but rather maintain current choices while planning subsequent choices. These data offer preliminary evidence of rapid heuristic-based computations in rd-mPFC and the hippocampus during sequential planning that might further elucidate how we make inferences about states beyond a current subjective state [88].

## Materials and Methods

### Participants

Thirty-four healthy adult participants performing the fMRI experiment gave informed written consent and were studied and compensated (as approved by the local research ethics committee at University College London and in accordance with Declaration of Helsinki protocols). Due to poor participant performance (answering less than 75% of trials correctly) in the fMRI experiment, we removed five participants, leaving a final sample of 29 participants (14 female; 23.4 mean age in y; SD of 4.09 y). All participants were right-handed had normal or corrected-to-normal vision and reported good health with no prior history of neurological disease.

### Task

Stimuli were presented using the Cogent (http://www.vislab.ucl.ac.uk/cogent.php) toolbox running in MATLAB (Mathworks, Natick, MA, USA). Over the course of 220 trials, participants viewed 220 different mazes from a slightly tilted (overhead) viewpoint and later chose from first-person viewpoints within mazes generated using Blender (http://www.blender.org). All mazes had a starting location (a red square) towards the bottom of the maze and a goal location (a green square) further into the maze. Mazes differed by hierarchical depth (number of paths to a goal location): there were 110 mazes with two possible routes (shallow mazes) and 110 mazes with four possible routes (deep mazes).

In the scanner, participants were first presented with pictures of mazes of varying difficulty (from our overhead viewpoint) and then asked to determine the shortest path from a starting location (a red square) at the bottom of the screen to the goal location (a green square). The overhead view appeared on the screen for 3.25 s, after which a location (choice point) along the path was highlighted briefly for 250 ms with an orange circle. The choice point location could either be the starting location or, if there were four paths to the goal location, a second choice point. Crucially, participants would only have to make a decision about one choice point for each trial. At any choice point, it was necessary to choose between two different directions, which could be left, forward, or right, with an additional option to select equal, if both routes were the same distance. No second choice points with two incorrect choices were ever chosen, only a second choice point along the optimal path after the starting location could be chosen (due to viewpoint constraints, only 47 choice points further were chosen versus the initial starting point/red square, which was chosen 53 times). After the choice point was highlighted, a “zoomed in” viewpoint of this location (always one square back and facing the same direction as the overhead viewpoint) was presented. Depending on the possible direction at the location, participants had less than 1,500 ms to decide whether to go left, forward, right, or occasionally either direction. If no button press was made within 1,500 ms, the trial counted as an incorrect trial and the experiment moved on to the 1500-ms intertrial interval (ITI) phase. Participants never received any feedback or reward for making the correct choice. As soon as participants chose a direction, the ITI phase of a trial began. Participants repeated this trial sequence 110 times per session, for a total of two sessions. Sessions lasted approximately 10–15 min. Session order was counterbalanced between participants.

All participants completed a brief practice session consisting of 40 mazes/trials before the experiment (on a laptop outside of the scanner). Deep mazes contained another branch/choice between routes further in the maze, and the path length to reach the two choice points further in the maze was always equal. Mazes had square tiled floors and were 8 x 8, 9 x 9, or 10 x 10 squares in total area. In shallow mazes, path length differences were split between 2, 4, and 6, with one catch trial per session having equal path lengths. In deep mazes, path length differences were split between 2 (small difference), 4 (medium difference), or 6 (large difference) squares (for an example, see square tiles in the mazes presented in Fig 1) for the two paths at the starting location and a path length difference of 2, 4, or 6 squares at the optimal choice point in the maze. There was one catch trial for deep and shallow mazes in each session, each containing all equal path lengths (path length differences of 0). In sum, shallow trials could either have path length difference of 2,4, and 6, while deep maze trials could be 2, 2; 2, 4; 2, 6; 4, 2; 4, 4; 4, 6; 6, 2; 6, 4; 6, 6; (e.g. 4, 2 would have a medium path length difference of 4 at the starting location, whereas the second choice point would have a small path length difference of 2; see Fig 1C for examples).

### fMRI Acquisition

Functional images were acquired on a 3T Siemens Trio scanner. BOLD T2*-weighted functional images were acquired using a gradient-echo EPI pulse sequence acquired obliquely at 45° with the following parameters: repetition time, 3,360 ms; echo time, 30 ms; slice thickness, 2 mm; inter-slice gap, 1 mm; in-plane resolution, 3 × 3 mm; field of view, 64 × 72 mm^2^; 48 slices per volume. A field-map using a double echo FLASH sequence was recorded for distortion correction of the acquired EPI [89]. After the functional scans, a T1-weighted 3-D MDEFT structural image (1 mm^3^) was acquired to co-register and display the functional data.

### fMRI Analysis

Functional images were processed and analysed using SPM8 (www.fil.ion.ucl.uk/spm). The first five volumes were discarded to allow for T1 equilibration. Standard preprocessing included correction for differences in slice acquisition timing, realignment/unwarping to correct for inter-scan movement, and normalization of the images to an EPI template (specific to our sequence and scanner) that was aligned to the T1 Montreal Neurological Institute (MNI) template. Finally, the normalized functional images were spatially smoothed with an isotropic 8-mm full-width half maximum Gaussian kernel. For the model described below, all regressors, with the exception of six movement parameters of no interest, were convolved with the SPM hemodynamic response function. Data were also high-pass filtered (cut-off period = 128 s). Statistical analyses were performed using a univariate GLM with a rapid event-related experimental design.

GLM1 was based on path length differences (see task description for possible path length differences): for the two shortest paths present at the starting point (Initial Path Length Difference), the path length difference between the shortest path and the longest unchosen path length that was not available at the second choice point (Unchosen Path Length Difference), the path length difference at the second choice point (Prospective Path Length Difference), log RT for the subsequent decision phase (Log RT), length of the shortest available path (Length of the Shortest Path), whether the participant made a correct choice during the subsequent choice phase (Performance), and whether the first or second choice point was prompted for deep maze trials (see Fig 1D for schematic showing the paths contributing to Initial Path Length Difference, Prospective Path Length Difference, and Unchosen Differences). For shallow trial regressors, there were only parametric regressors for Initial Path Length Difference, Log RT, Length of the Shortest Path, and Performance. Inferences about the effects of uncertainty were based upon *t* tests using the standard summary statistic approach for second-level random effects analysis (see S1 Text for additional follow-up GLMs and corresponding results and S5 Table for a complete table of conditions and parametric regressors for each GLM).

We conducted a PPI analysis [15] to examine hippocampal coupling with rd-mPFC and the rest of the brain during deep versus shallow planning trials. The group-level right posterior hippocampus peak (x = 24, y = −37, z = −8) that correlated with increased RT served as a centre for the spherical region of interest (8-mm radius). The first eigenvariate from these ROIs constituted the physiological variable. The psychological variable was the contrast vector representing the task effect of deep versus shallow mazes. These regressors and their interaction term were estimated at the first level. Contrast images associated with the PPI regressor were then entered into a one-sample *t* test.

Post hoc statistical analyses were conducted using 8-mm radius spheres in MarsBar [90] toolbox within SPM8 around the respective peak voxel specified in the GLM analysis. This allowed us to compare the effects of different parametric regressors of interest (e.g., to determine whether a length of the shortest available path effect was present in a region defined by an orthogonal main effect of prospective path length difference). This ensured we did not make any biased inferences in our post hoc analyses.

Given the previously hypothesized role of the hippocampus in spatial planning, we report whether hippocampal peak-voxels survive SVC for multiple comparisons (*p* < 0.05) based on a bilateral ROI of the hippocampus constructed using the SPM Anatomy toolbox [91–92]. For all analyses outside of the hippocampus, we report activations surviving an uncorrected statistical threshold of *p =* 0.005 and cluster-level correction for multiple comparisons (FWE *p* < 0.05), unless indicated otherwise. We also mention whether any significant clusters had a very large cluster extent (k > 2,000), and the cluster extent for every significant effect is reported in S7–13 Tables. Coordinates of brain regions are reported in MNI space. BOLD signal time courses in S5 Fig were plotted using the rfxplot toolbox [93].

## Supporting Information

S1 Text Supporting results and methods. (DOCX)

S1 Fig Prospective choice behaviour. (A) Accuracy when participants are prompted on the prospective/second choice point further in the maze. Left: Fraction of correct prospective choices split by whether there was a small, medium, or large path length difference at the prospective choice point. Right: Fraction of correct prospective choices split by whether there was a small, medium, or large path length difference between the two shortest options available at the first initial choice point. (B) Log RT when participants are prompted on a choice point further in the maze. Left: Log RT for prospective choices split by whether there was a small, medium, or large path length differences at the second choice points. Right: Log RT for prospective trials split by whether there was a small, medium, or large path length difference between the two shortest options available at the first choice point.
(PDF)

S2 Fig Large versus small prospective path length differences. Anterior prefrontal peak for initial (in shallow mazes) versus prospective path length differences. Images showing dlPFC peak of cluster (circled in blue) also containing rd-mPFC sub-peak that significantly responded to small prospective versus small path length differences in shallow mazes (see Fig 3E). dlPFC cluster survives FWE correction *p* < 0.05 for multiple comparisons and is shown at *p* < 0.005 uncorrected.
(PDF)

S3 Fig Response to initial choice path length differences. Top: Sagittal image centred on vmPFC activation that responded to large initial path length differences. Regions responding to large initial path length differences shown in orange and regions responding to small initial path length differences shown in blue. Bottom: Effect size for 8-mm sphere around the vmPFC peak for initial, prospective, and unchosen path length differences. Asterisk signifies *p* < 0.05.
(PDF)

S4 Fig Responses to proximal shortest available path length. Images centred on rd-mPFC that correlated with smaller distance between the starting and goal location (i.e., length of the shortest available path).
(PDF)

S5 Fig dACC activation during planning preceding incorrect choices. (A) Left: Sagittal image showing dACC/pSMA activity during planning that was higher prior to incorrect versus correct choice trials. Right: Effect size for an 8-mm sphere around the dACC/pSMA peak voxel showing that there is no significant difference (*p* < 0.05) in the correlation with subsequently incorrect choices for deep versus shallow planning trials (mean ± SEM). Asterisks indicate a significant correlation (*p* < 0.05) with path length differences. A negative effect size represents a correlation with incorrect trials, whereas a positive effect size represents a correlation with correct trials. (B) Evoked dACC/pSMA BOLD response during planning separated by subsequently correct and incorrect choice trials (mean ± SEM).
(PDF)

S6 Fig Deep versus shallow planning interactions. (A) Images centred on PPC region that significantly responded to smaller initial path length differences in shallow versus deep mazes. (B) Images centred on left PPC region that significantly responded to subsequently incorrect choices in shallow versus deep mazes. (C) Images centred on right inferior temporal cortex region that responded to increasing distance between the starting and goal location in deep versus shallow mazes.
(PDF)

S1 Table Bayesian model comparison of reduced forms of the behavioural model in Equation 1. The first and second columns report the model variant and the corresponding Bayesian information criterion (BIC; summed across participants). A small BIC reflects greater (log) model evidence.
(DOCX)

S2 Table Shannon entropy values (H) by path length difference. Several models of RT were compared using the BIC after penalizing for the number of parameters. A smaller BIC value indicates that a model has higher evidence, after penalizing its accuracy for its complexity or number of parameters.
(DOCX)

S3 Table Mean probability of making the correct choice using our Shannon entropy model. (DOCX)

S4 Table GLM1. Model-free regressors.
(DOCX)

S5 Table GLM2. Shannon entropy model-based regressors.
(DOCX)

S6 Table GLM3. PPI correct or incorrect choice regressors.
(DOCX)

S7 Table Initial path length differences. List of peak voxels for clusters found in the initial path length difference contrast. Please note that despite our stringent threshold (*p* < 0.005 activation threshold, cluster-based threshold *p* < 0.05), many of the activations are very large (k > 2,000) and span multiple brain regions. Consequently, the labels assigned to each cluster should be interpreted with caution.
(DOCX)

S8 Table Prospective path length differences. List of peak voxels for clusters found in the prospective path length difference contrast. Please note that despite our stringent threshold (*p* < 0.005 activation threshold, cluster-based threshold *p* < 0.05), some activations are very large (k > 2,000) and span multiple brain regions. Consequently, the labels assigned to each cluster should be interpreted with caution.
(DOCX)

S9 Table Unchosen path length differences. List of peak voxels for clusters found in the Unchosen Path Length difference contrast.
(DOCX)

S10 Table Planning responses related to subsequent choice RT. List of peak voxels for clusters found in subsequent choice RT contrast. Please note that despite our stringent threshold (*p* < 0.005 activation threshold, cluster-based threshold *p* < 0.05), some activations are very large (k > 2,000) and span multiple brain regions. Consequently, the labels assigned to each cluster should be interpreted with caution.
(DOCX)

S11 Table Length of shortest available path. List of peak voxels for clusters found in the length of shortest available path contrast. Please note that despite our stringent threshold (*p* < 0.005 activation threshold, cluster-based threshold *p* < 0.05), some activations are very large (k > 2,000) and span multiple brain regions. Consequently, the labels assigned to each cluster should be interpreted with caution.
(DOCX)

S12 Table Accuracy. List of peak voxels for clusters found in the accuracy contrast.
(DOCX)

S13 Table Deep V shallow interactions. List of peak voxels for clusters found in contrasts for deep versus shallow interactions related to different parametric regressors. Please note that despite our stringent threshold (*p* < 0.005 activation threshold, cluster-based threshold *p* < 0.05), some of the activations are very large (k > 2,000) and span multiple brain regions. Consequently, the labels assigned to each cluster should be interpreted with caution.
(DOCX)

S1 Data Data underlying plots in Fig 2 (panels A and B).
(XLS)

S2 Data Data underlying plots in Fig 3 (panels C and D).
(XLS)

S3 Data Data underlying plots in Fig 4 (panel A).
(XLS)

S4 Data Data underlying plots in Fig 5 (panels C and D).
(XLS)

## Abbreviations

BOLD: blood-oxygen-level dependent
dACC: dorsal anterior cingulate cortex
dlPFC: dorsolateral prefrontal cortex
fMRI: functional magnetic resonance imaging
FPC: frontopolar cortex
FWE: family-wise error
GLM: general linear model
IPS: intraparietal sulcus
ITI: intertrial interval
lFPC: lateral frontopolar cortex
LOC: lateral occipital cortex
MNI: Montreal Neurological Institute
PCC: posterior cingulate cortex
PFC: prefrontal cortex
pgACC: pregenual anterior cingulate cortex
PMC: premotor cortex
PPC: posterior parietal cortex
PPI: psychophysiological interaction
pSMA: pre-supplementary motor area
rd-mPFC: rostrodorsal medial prefrontal cortex
ROI: region of interest
RT: reaction time
SD: standard deviation
SEM: standard error of the mean
SFG: superior frontal gyrus
SVC: small-volume correction
vmPFC: ventromedial prefrontal cortex
