# Supplementary material for: The Neural Representation of Prospective Choice during Spatial Planning and Decisions
Source: PLoS Biol. 2017 Jan 12;15(1):e1002588. doi: 10.1371/journal.pbio.1002588 (PMC5231323; doi:10.1371/journal.pbio.1002588)
Supplement: S1 Text — (DOCX) [file pbio.1002588.s001.docx]

**S1 Text. Supporting Results and Methods.**

**Supplementary Results**

We quantified uncertainty during planning by fitting a formal model based on differences in the length of available paths (see Equation 1). Specifically, we characterized choice uncertainty using the entropy of choice probabilities based on a softmax function of path lengths. High entropy corresponds to uncertainty induced by similar path lengths. To estimate the requisite softmax (sensitivity or precision) parameter, we assumed that reaction times reflect uncertainty about each choice. This allowed us to model (log) reaction times in terms of entropy (H) over trials (and participants) using a simple linear regression model. The ensuing behavioral modeling provided an estimate of the precision parameter (B) and associated measure of trial-specific uncertainty for each participant:

*$\begin{matrix} \log(RT)=RT_{0}+k\cdot H[P(s)]+error \\ H[P(s)]=-\sum_{i} P(s_{i})\cdot\log P(s_{i}) \\ P(s_{i})=\frac{\exp(-B\cdot l_{i})}{\sum_{i} \exp(-B\cdot l_{i})} \end{matrix}$*

***Equation 1:*** *P(s): probability of choosing the i-th path (s_i_) as the shortest of all possible path lengths for a trial; RT_0_: intercept across all trials; l_i_: potential path length; H: Shannon Entropy; k: slope across all trials; B: precision or inverse temperature across all trials; RT: reaction time for a given trial.*

This implicit computational model of choice behavior provided trial-specific measures of choice uncertainty (H) that enabled us to identify its fMRI correlates.

*Computational Model and Behavior*

We used the Bayesian Information Criterion (BIC) [1] to compare reduced versions of the computational model (Equation 1). Entropy-based predictions of RT were best characterized using a restricted precision parameter (*B*) between 0 and 2. Quantitatively, a precision of two or less meant that the probability of choosing the shortest path was less than ~99% for most trials. The model with a restricted precision had greater evidence than variants of the model without constraints and more constrained versions, where some of the parameters were fixed (see S1 Table). These alternative models included models without an effect of entropy (*k*=0), an intercept only model, and a null model with just random effects.

We used the mean precision parameter (B), over participants to compute trial-specific entropies (uncertainty; H value from Eq 1) as predictor variables in our neuroimaging analyses [2-4] (See S2 Table for mean probability of making a correct choice with a given path length difference). As expected, mean entropy values (*H*) correlated with mean log reaction time within participants (t(28)=13.0; p<.001); i.e. trials with greater uncertainty had longer reaction times.

*Additional Functional Magnetic Resonance Imaging Analyses*

In a supplementary fMRI analyses, we used a standard general linear model (GLM2 – *model based*) comprising regressors for deep and shallow maze planning phases, inter-trial intervals (ITI), and seven parametric effects including choice uncertainty (entropy), length of the shortest path, and whether the participant subsequently made the correct choice (performance) separately for both deep and shallow maze planning – with an additional parametric effect in deep mazes for prospective uncertainty (see S4 Table for all conditions and related parametric regressors). In deep mazes, prospective uncertainty (entropy) was based on the path lengths available from the second choice point of the shortest path, while choice uncertainty was based on all four paths (Fig 1). In the group-level analyses presented below, we report one-sample or paired t-tests of parametric effects.

Modeling the effects of uncertainty, length of the shortest path to the goal, and performance separately (for deep and shallow mazes), allowed us to examine the interaction between our parametric effects and depth of planning. Consequently, we were able to ask whether the number of available choices affected uncertainty, shortest path length, and performance -dependent neuronal responses. In what follows, we report the simple effects of choice, prospective uncertainty, shortest path length, and performance respectively. We also conducted *post hoc* (region of interest) analyses to characterize the interaction of prospective versus shallow choice uncertainty in the areas showing simple main effects.

*Choice Uncertainty*

We tested for the main effect of choice uncertainty on all planning phase trials. We found significant effects of uncertainty in ventral mPFC (vmPFC, x=6;y=35;z=-11; Z-score: 5.18), along with another very large cluster in the posterior cingulate cortex (PCC: x=-3;y=-46;z=37; Z-score: 5.02), that responded to decreasing choice uncertainty (i.e. larger path length differences) and survived cluster-level family wise error (FWE) correction (p<.05) for multiple comparisons across the whole brain.

Conversely, responses in the dorsal anterior cingulate cortex/pre-supplementary motor area (dACC/pSMA; x=9;y= 20; z=43; Z-score: 5.81), bilateral posterior parietal cortex (right PPC; x=21; y=-64; z=55; Z-score: 4.92; left PPC x=-9; y=-70; z=52;Z-score: 4.68), left dorsolateral PFC (x=-18, y=2, z=58; Z-score: 4.05), and right insula (x=30;y=20;z=1;Z-score: 4.83) increased with choice uncertainty (i.e. smaller path length differences) and survived whole-brain cluster-level family wise error (FWE) correction (p<.05) at a single voxel threshold of p<.005 uncorrected.

*Prospective Uncertainty*

We then asked whether, during deep maze trials, there were fMRI responses specifically related to inferences about the second choice point; i.e., BOLD changes selectively related to choosing between the two paths at the second choice point that were not explained by uncertainty about all available paths. We found significant effects in the dorsal posterior/mid-cingulate cortex (dPCC/MCC: x=9;y=-19;z=49; Z-score:4.61) and the right insula (x=30;y=-10;z=7; Z-score: 4.09) with a peak in rostrodorsal mPFC/superior frontal gyrus at the border of BA8 and 9 (rd-mPFC: x=-15;y=35;z=37; Z-score: 3.17; peak voxel from GLM1 also had the same Z-score), and a separate cluster in left lateral frontopolar cortex (FPC: x=-27;y=56;z=7; Z-score: 3.75). Notably, the rd-mPFC/superior frontal gyrus region responding to prospective uncertainty covered the area located between the dACC/pSMA region that responded most significantly to increasing choice uncertainty and the rostral mPFC cluster that responded to decreasing choice uncertainty. We additionally found significant clusters in the left inferior parietal lobule (x=-54;y=-70;z=13; Z-score: 4.57) and portions of right angular gyrus/TPJ (x=45;y=-40;z=10; Z-score: 4.62) that also increased with prospective uncertainty. All clusters were significant for cluster-level FWE correction (p<.05) for multiple comparisons at a single voxel threshold of p<.005 uncorrected. We did not observe any significant effect of decreasing prospective uncertainty.

To characterize the above prospective uncertainty effects in the PFC in more detail, we then examined which of these regions exhibited significantly greater responses for increasing prospective versus shallow choice uncertainty. By comparing the responses in this way, we hoped to characterize the functional specialization of the key prefrontal areas identified above– avoiding problems related to large cluster extents – and identify any region that showed a significantly greater effect of uncertainty for subsequent/prospective choices, relative to initial choices. Paired t-tests for prospective versus shallow uncertainty revealed that all regions we observed responding to increasing prospective uncertainty: dorsal PCC/MCC (nearest observed peak x=6;y=-28;z=52; Z-score:5.52), right insula (nearest observed peak x=30;y=-7;z=4; Z-score:5.07), rd-mPFC (nearest observed sub-peak x:-12;y=38; z=43; Z-score:3.62), lFPC (nearest observed sub-peak in the frontal pole: x=-15, y=53;z=13; Z-score:3.95), left IPL (nearest observed sub-peak x=-51;y=-67;z=16; Z-score:4.60), and right angular gyrus/TPJ (same peak coordinate: Z-score:4.83) were part of clusters that exhibited significantly greater responses for increasing prospective versus shallow choice uncertainty FWE cluster-corrected p<.05 at the whole-brain level. Notably, PCC and right angular gyrus/TPJ were the only regions that responded significantly to both increasing prospective choice uncertainty and decreasing choice uncertainty at the whole-brain level.

Given our interest in medial prefrontal contributions to planning, we then determined whether our rd-mPFC prospective uncertainty effect was present across mPFC, or was specific to rd-mPFC. We examined the neighboring caudal dmPFC region that significantly responded to increasing choice uncertainty, dACC/pSMA (x=9;y= 20; z=43). Converse to rd-mPFC, the main effect of regressor in dACC/pSMA was driven by smaller responses to prospective uncertainty relative to shallow choice uncertainty (t(28)=2.50; p=.019). We then investigated the vmPFC region that responded to decreasing choice uncertainty and found that, similar to rdmPFC, responses were significantly higher for prospective uncertainty (t(28)=2.72; p=.011). However the effect in vmPFC was driven by responses to decreasing choice uncertainty without any significant response to prospective uncertainty, rather than increasing prospective uncertainty like rd-mPFC. Notably, this finding differs from our path length difference analysis, where we observed vmPFC/pgACC responses to increasing initial and prospective path length differences. This discrepancy is possibly due to the inclusion of all path length differences for our choice uncertainty calculation, whereas only the two shortest paths available at the starting choice point informed initial path length differences.

**Supplementary Methods**

*Computational Model*

We used a trial-by-trial computational model of participants’ reaction times in order to quantify how different path lengths determined participants’ uncertainty during planning. Uncertainty (or inverse precision) was measured using Shannon entropy [5](Eq.1). Shannon entropy (*H*) was calculated by estimating the distribution of choice probabilities using a softmax function of path length (see [4] for a summary trial-by-trial data analysis using computational models). The inverse temperature parameter (*B*) of the softmax function was optimized using each participant's log reaction times. *B* was restricted between 0 and 2, since values above 2 would lead to choice probabilities of >99% for making the correct choice for all trials. We constructed predictions of neuronal responses in terms of the Shannon entropy (*H*) of the choice probability in each trial for fMRI (see Eq. 1).

We optimized all participant-specific parameters across trials using maximum likelihood estimation and the optimization toolbox in MATLAB (MathWorks, Inc). We then calculated trial-by-trial parameter estimates of *H* (choice uncertainty) using the group average softmax (precision or inverse temperature) parameter. The ensuing trial-by-trial entropy measures were then used to predict BOLD responses in our supplementary neuroimaging analyses. Prospective entropy/uncertainty for the second choice point was generated from using the group average softmax function of the two path lengths available at the second choice point.

GLM2 (*model-based*): There were two periods of interest, the 3.25s planning phase and 1.5s baseline intertrial-interval (ITI) conditions for both deep and shallow mazes, which were modeled as boxcar functions and convolved with a canonical hemodynamic response function (HRF) to create regressors of interest (constructed for all GLMs). For each planning phase regressor (deep and shallow maze trials) there were parametric regressors based on choice uncertainty, length of the shortest available path, and performance (1=incorrect choice; 2= correct choice). There was also an additional parametric regressor for deep trials for prospective uncertainty/entropy (H) between the two path lengths at the second choice point (see S4 Table for a complete table of parametric regressors and conditions). Inferences about the effects of uncertainty were based upon t-tests using the standard summary statistic approach for second level random effects analysis (S4 Table).

GLM 3 (model-free/*performance-based*): In order to assay performance related differences in the PPI analysis, a GLM was constructed where there were two conditions of interest, the 3.25 s planning phase and 1.5s baseline intertrial-interval (ITI) conditions split into regressors for correct and incorrect trials in both deep and shallow mazes (S7 Table).

**Supplementary References**

1. Schwarz, G. Estimating the dimension of a model. Ann Stats. 1978;6: 461-464.

2. Daw ND, O’Doherty JP, Dayan P, Seymour B, Dolan RJ. Cortical substrates for exploratory decisions in humans. Nature. 2006;441: 876-879.

3. Gläscher J, Daw N, Dayan P, O’Doherty JP. States versus rewards: dissociable neural prediction error signals underlying model-based and model-free reinforcement learning. Neuron. 2010;66: 585-595.

4. Daw, N.D. in Decision Making, Affect, and Learning: Attention and Performance XXIII, eds:Delgado MR, Phelps EA, Robbins TW (Oxford Univ Press, Oxford) (2011).

5. Shannon CE. A mathematical theory of communication. The Bell System Technical Journal. 1948;27: 379-423.

6. Kass RE, Raftery AE. Bayes factors. J Amer Stat Assoc.1995;90: 773-795.
