# Supplementary figures and images for: The Neural Representation of Prospective Choice during Spatial Planning and Decisions
Source: PLoS Biol. 2017 Jan 12;15(1):e1002588. doi: 10.1371/journal.pbio.1002588 (PMC5231323; doi:10.1371/journal.pbio.1002588)

A

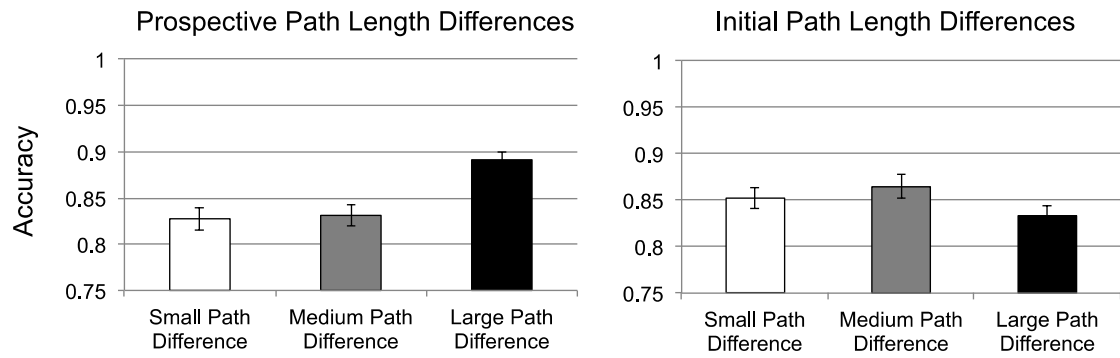

B

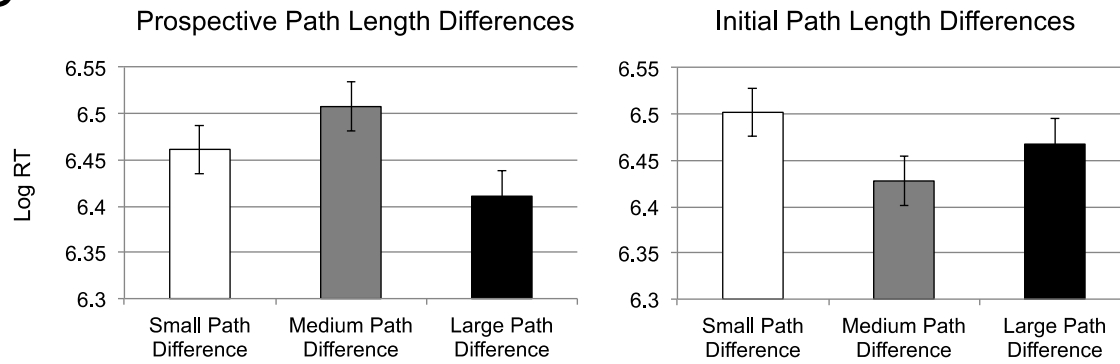

Supplement: S1 Fig — (A) Accuracy when participants are prompted on the prospective/second choice point further in the maze. Left: Fraction of correct prospective choices split by whether there was a small, medium, or large path length difference at the prospective choice point. Right: Fraction of correct prospective choices split by whether there was a small, medium, or large path length difference between the two shortest options available at the first initial choice point. (B) Log RT when participants are prompted on a choice point further in the maze. Left: Log RT for prospective choices split by whether there was a small, medium, or large path length differences at the second choice points. Right: Log RT for prospective trials split by whether there was a small, medium, or large path length difference between the two shortest options available at the first choice point. (PDF) [file pbio.1002588.s002.pdf]

Large v. Small Prospective  
Path Length Differences

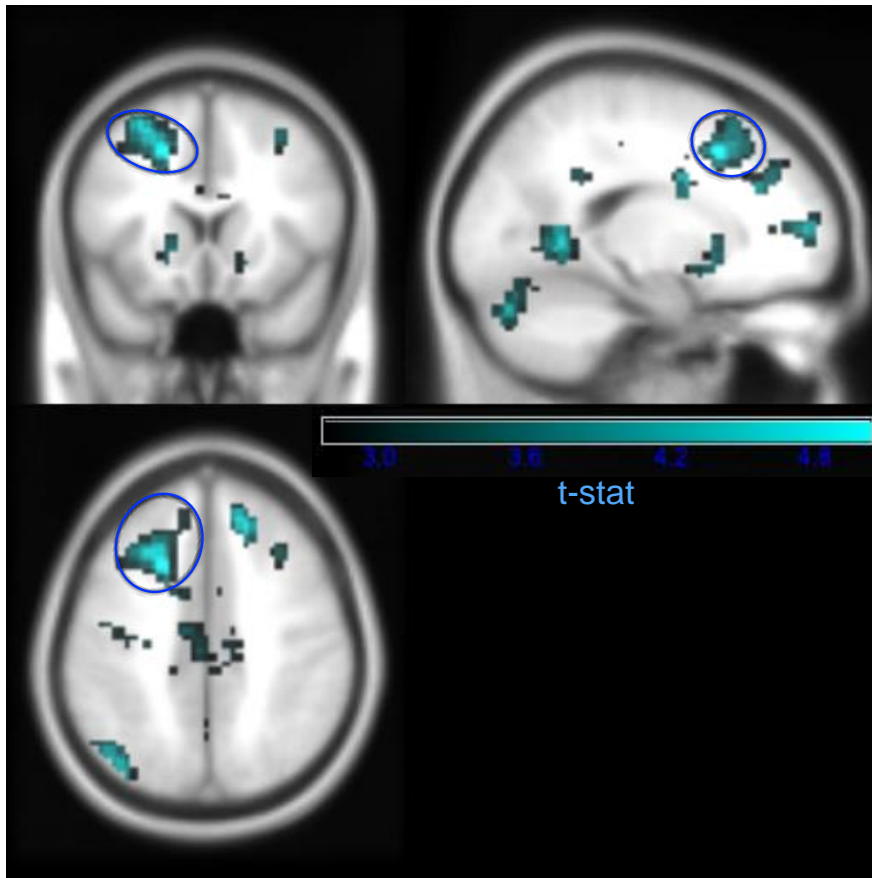

Supplement: S2 Fig — Anterior prefrontal peak for initial (in shallow mazes) versus prospective path length differences. Images showing dlPFC peak of cluster (circled in blue) also containing rd-mPFC sub-peak that significantly responded to small prospective versus small path length differences in shallow mazes (see Fig 3E). dlPFC cluster survives FWE correction p < 0.05 for multiple comparisons and is shown at p < 0.005 uncorrected. (PDF) [file pbio.1002588.s003.pdf]

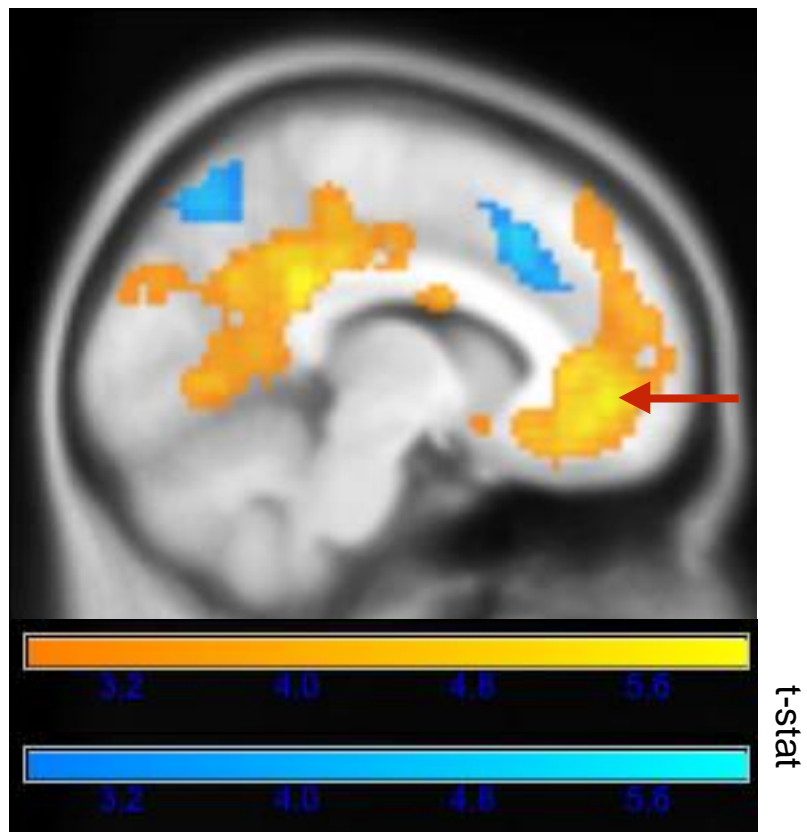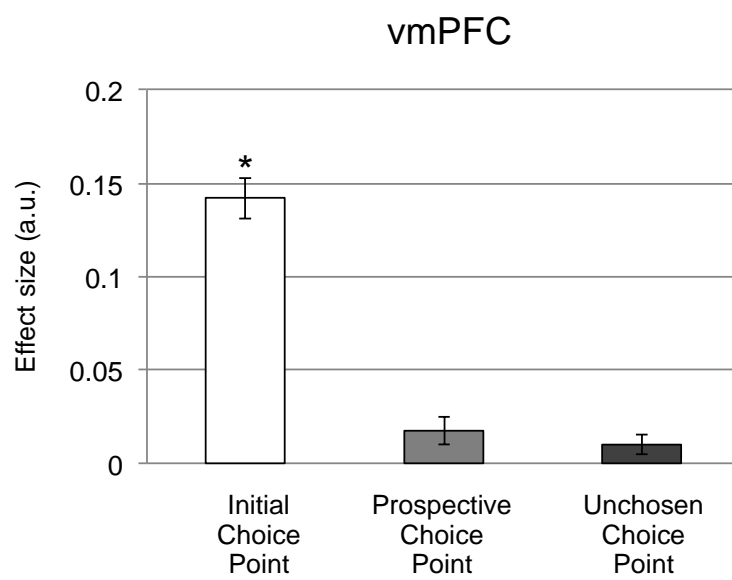

Supplement: S3 Fig — Top: Sagittal image centred on vmPFC activation that responded to large initial path length differences. Regions responding to large initial path length differences shown in orange and regions responding to small initial path length differences shown in blue. Bottom: Effect size for 8-mm sphere around the vmPFC peak for initial, prospective, and unchosen path length differences. Asterisk signifies p < 0.05. (PDF) [file pbio.1002588.s004.pdf]

# Decreasing Length of the Shortest Path

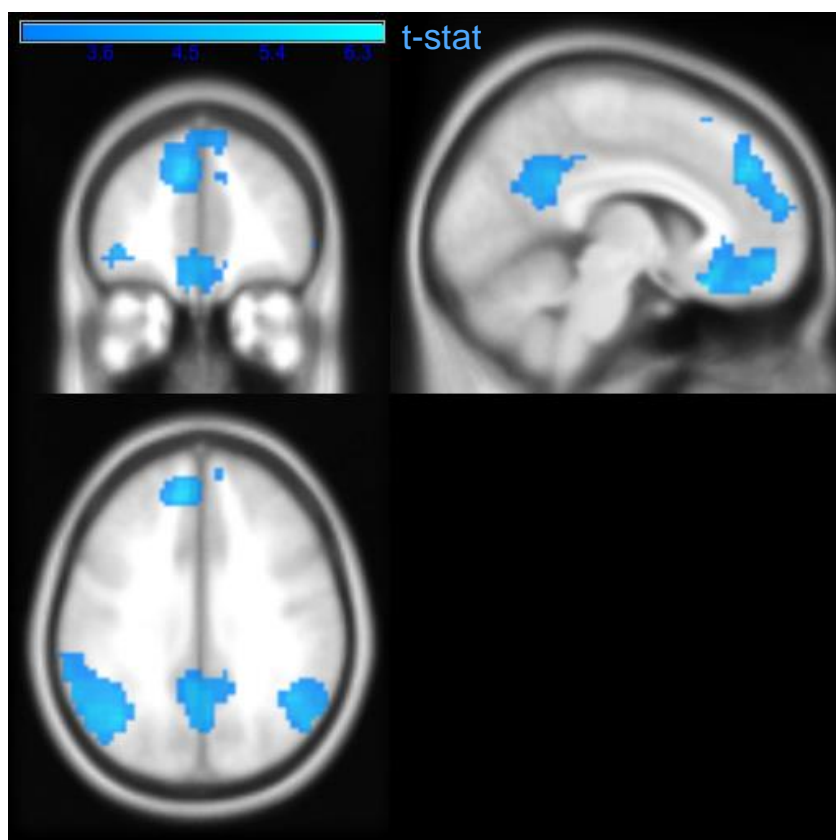

Supplement: S4 Fig — Images centred on rd-mPFC that correlated with smaller distance between the starting and goal location (i.e., length of the shortest available path). (PDF) [file pbio.1002588.s005.pdf]

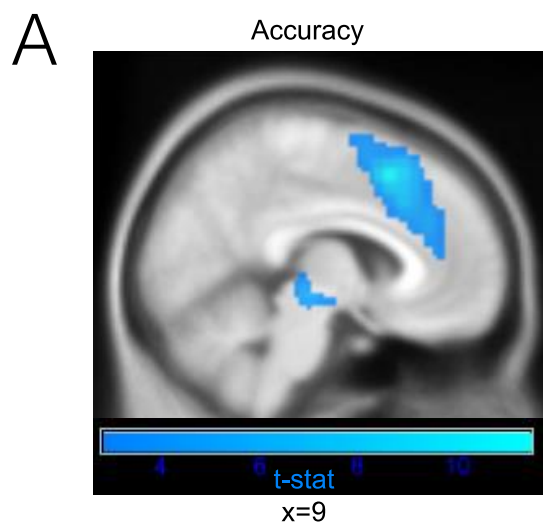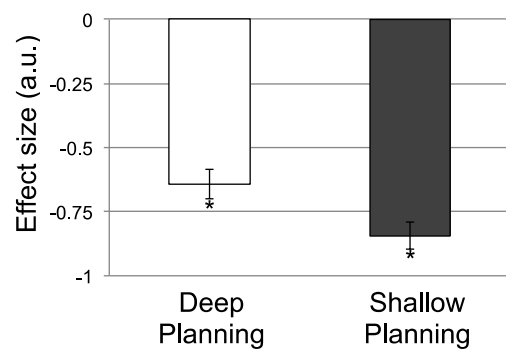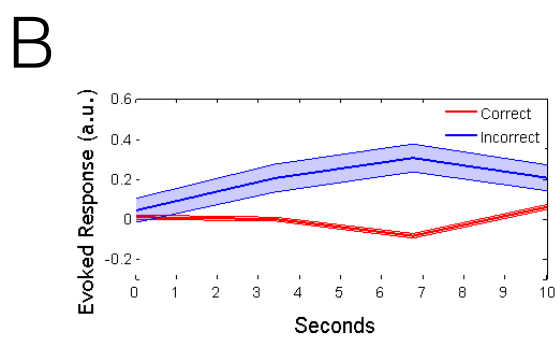

Supplement: S5 Fig — (A) Left: Sagittal image showing dACC/pSMA activity during planning that was higher prior to incorrect versus correct choice trials. Right: Effect size for an 8-mm sphere around the dACC/pSMA peak voxel showing that there is no significant difference (p < 0.05) in the correlation with subsequently incorrect choices for deep versus shallow planning trials (mean ± SEM). Asterisks indicate a significant correlation (p < 0.05) with path length differences. A negative effect size represents a correlation with incorrect trials, whereas a positive effect size represents a correlation with correct trials. (B) Evoked dACC/pSMA BOLD response during planning separated by subsequently correct and incorrect choice trials (mean ± SEM). (PDF) [file pbio.1002588.s006.pdf]

A

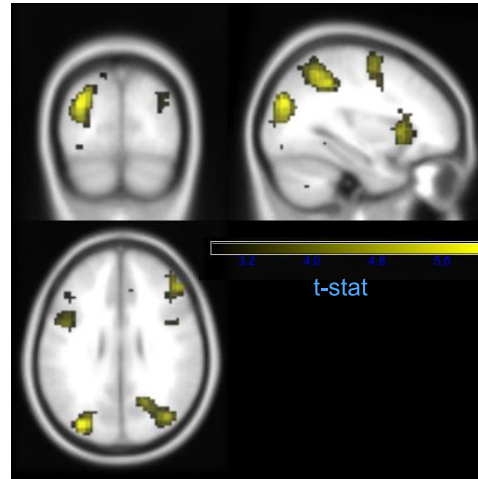

B

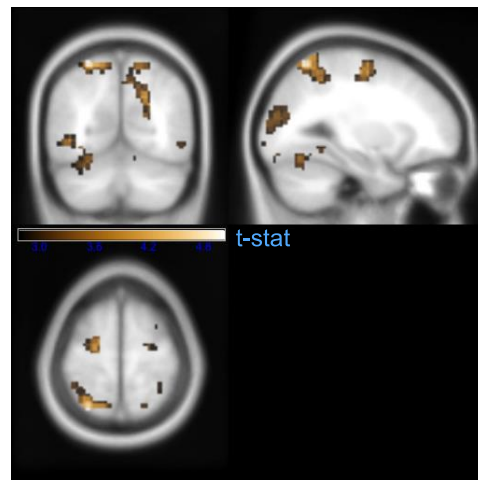

C

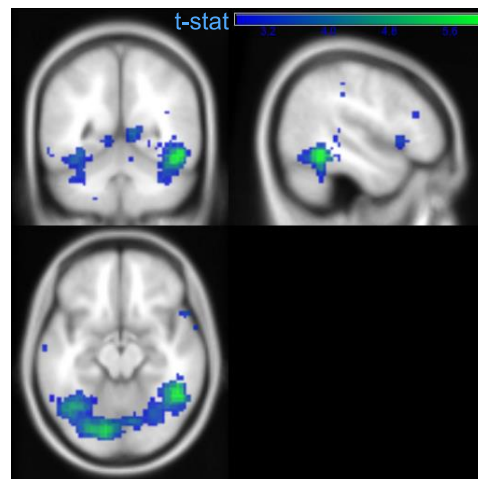

Supplement: S6 Fig — (A) Images centred on PPC region that significantly responded to smaller initial path length differences in shallow versus deep mazes. (B) Images centred on left PPC region that significantly responded to subsequently incorrect choices in shallow versus deep mazes. (C) Images centred on right inferior temporal cortex region that responded to increasing distance between the starting and goal location in deep versus shallow mazes. (PDF) [file pbio.1002588.s007.pdf]
