# Supplementary material for: The Neural Representation of Prospective Choice during Spatial Planning and Decisions
Source: PLoS Biol. 2017 Jan 12;15(1):e1002588. doi: 10.1371/journal.pbio.1002588 (PMC5231323; doi:10.1371/journal.pbio.1002588)
Supplement: S1 Table — The first and second columns report the model variant and the corresponding Bayesian information criterion (BIC; summed across participants). A small BIC reflects greater (log) model evidence. (DOCX) [file pbio.1002588.s008.docx]

**S1 Table**

| Model variant | BIC |
| --- | --- |
| *Restricted precision model* | *9343 |
| *Intercept only model* | 9638 |
| *Null (random effects) model* | 41554 |
| *Full Model (all free parameters)* | 82814 |
| *Null (no Entropy) entropy* | 109838 |
