# Supplementary material for: The Neural Representation of Prospective Choice during Spatial Planning and Decisions
Source: PLoS Biol. 2017 Jan 12;15(1):e1002588. doi: 10.1371/journal.pbio.1002588 (PMC5231323; doi:10.1371/journal.pbio.1002588)
Supplement: S2 Table — Several models of RT were compared using the BIC after penalizing for the number of parameters. A smaller BIC value indicates that a model has higher evidence, after penalizing its accuracy for its complexity or number of parameters. (DOCX) [file pbio.1002588.s009.docx]

**S2 Table**

| Path Length Difference | Mean H Value |
| --- | --- |
| 2 | 0.65 |
| 4 | 0.54 |
| 6 | 0.41 |
| 2-2 (Deep) | 1.3 |
| 2-4 (Deep) | 1.21 |
| 2-6 (Deep) | 1.14 |
| 4-2 (Deep) | 1.21 |
| 4-4 (Deep) | 1.15 |
| 4-6 (Deep) | 1.14 |
| 6-2 (Deep) | 1.09 |
| 6-4 (Deep) | 1.04 |
| 6-6 (Deep) | 0.92 |
