# Supplementary material for: The Neural Representation of Prospective Choice during Spatial Planning and Decisions
Source: PLoS Biol. 2017 Jan 12;15(1):e1002588. doi: 10.1371/journal.pbio.1002588 (PMC5231323; doi:10.1371/journal.pbio.1002588)
Supplement: S3 Table — (DOCX) [file pbio.1002588.s010.docx]

**S3 Table**

| Path Length Differences (Initial Path Difference/Prospective Path Difference | Mean Probability of Choosing Shortest Path |
| --- | --- |
| Small (Shallow) | 64.6% |
| Medium (Shallow) | 76.9% |
| Large (Shallow) | 85.9% |
| Small-Small (Deep) | 41.5% |
| Small-Medium (Deep) | 48.6% |
| Small-Large (Deep) | 52.1% |
| Medium-Small (Deep) | 48.6% |
| Medium-Medium (Deep) | 55.8% |
| Medium-Large (Deep) | 56.1% |
| Large-Small (Deep) | 55.9% |
| Large-Medium (Deep) | 61.3% |
| Large-Large (Deep) | 70.4% |
