# Supplementary material for: The Neural Representation of Prospective Choice during Spatial Planning and Decisions
Source: PLoS Biol. 2017 Jan 12;15(1):e1002588. doi: 10.1371/journal.pbio.1002588 (PMC5231323; doi:10.1371/journal.pbio.1002588)
Supplement: S4 Table — Model-free regressors. (DOCX) [file pbio.1002588.s011.docx]

**S4 Table**

| Regressor | Parametric 1 | Parametric 2 | Parametric 3 | Parametric 4 | Parametric 5 | Parametric 6 | Parametric 7 |
| --- | --- | --- | --- | --- | --- | --- | --- |
| Deep Mazes | Initial Path Difference | Prospective Path Difference | Unchosen Path Difference | Log RT | Shortest Path Length | Accuracy | Prompted Choice |
| Shallow Mazes | Initial Path Difference | Log RT | Shortest  Path Length | Accuracy | - | - | - |
| ITI | - | - | - | - | - | - | - |
