# Supplementary material for: The Neural Representation of Prospective Choice during Spatial Planning and Decisions
Source: PLoS Biol. 2017 Jan 12;15(1):e1002588. doi: 10.1371/journal.pbio.1002588 (PMC5231323; doi:10.1371/journal.pbio.1002588)
Supplement: S5 Table — Shannon entropy model-based regressors. (DOCX) [file pbio.1002588.s012.docx]

**S5 Table**

| Regressor | Parametric1 | Parametric2 | Parametric3 | Parametric4 |
| --- | --- | --- | --- | --- |
| Deep Mazes | Choice Uncertainty | Prospective Uncertainty | Shortest Available Path Length | Performance |
| Shallow Mazes | Choice Uncertainty | Shortest Available Path Length | Performance | - |
| ITI | - | - | - | - |
