# Supplementary material for: The Neural Representation of Prospective Choice during Spatial Planning and Decisions
Source: PLoS Biol. 2017 Jan 12;15(1):e1002588. doi: 10.1371/journal.pbio.1002588 (PMC5231323; doi:10.1371/journal.pbio.1002588)
Supplement: S6 Table — PPI correct or incorrect choice regressors. (DOCX) [file pbio.1002588.s013.docx]

**S6 Table**

| **Regressor** |
| --- |
| Deep Correct |
| Deep Incorrect |
| Shallow Correct |
| Shallow Incorrect |
| ITI |
