# Supplementary material for: The Neural Representation of Prospective Choice during Spatial Planning and Decisions
Source: PLoS Biol. 2017 Jan 12;15(1):e1002588. doi: 10.1371/journal.pbio.1002588 (PMC5231323; doi:10.1371/journal.pbio.1002588)
Supplement: S7 Table — List of peak voxels for clusters found in the initial path length difference contrast. Please note that despite our stringent threshold (p < 0.005 activation threshold, cluster-based threshold p < 0.05), many of the activations are very large (k > 2,000) and span multiple brain regions. Consequently, the labels assigned to each cluster should be interpreted with caution. (DOCX) [file pbio.1002588.s014.docx]

**S7 Table**

| Region (Larger Path Differences) | MNI coordinates (xyz) | peak Z-score | Cluster-corrected p-value | Cluster size (k) |
| --- | --- | --- | --- | --- |
| Temporoparietal junction | 66 -37 28 | 5.2 | p<.001 | 2105 |
| Posterior cingulate cortex | -12 -40 31 | 5.02 | p<.001 | 2301 |
| Ventromedial prefrontal cortex | 9 38 1 | 4.99 | p<.001 | 4352 |
| Region (Smaller Path Differences) | MNI coordinates (xyz) | peak Z-score | Cluster- corrected p-value | Cluster size (k) |
| Dorsal anterior cingulate/pre-supplementary motor area | 9 17 49 | 4.91 | p<.001 | 339 |
| Dorsolateral prefrontal cortex | 48 35 25 | 4.78 | p=.009 | 223 |
| Anterior insula | 30 20 1 | 4.59 | p=.03 | 174 |
| Posterior parietal cortex | 21 -61 58 | 4.55 | p<.001 | 1623 |
