# Supplementary material for: The Neural Representation of Prospective Choice during Spatial Planning and Decisions
Source: PLoS Biol. 2017 Jan 12;15(1):e1002588. doi: 10.1371/journal.pbio.1002588 (PMC5231323; doi:10.1371/journal.pbio.1002588)
Supplement: S8 Table — List of peak voxels for clusters found in the prospective path length difference contrast. Please note that despite our stringent threshold (p < 0.005 activation threshold, cluster-based threshold p < 0.05), some activations are very large (k > 2,000) and span multiple brain regions. Consequently, the labels assigned to each cluster should be interpreted with caution. (DOCX) [file pbio.1002588.s015.docx]

**S8 Table**

| Region (Smaller Path Differences) | MNI coordinates (xyz) | peak Z-score | Cluster corrected p-value | Cluster size (k) |
| --- | --- | --- | --- | --- |
| Dorsal anterior cingulate/pre-supplementary motor area | 6 23 37 | 5.08 | p<.001 | 3259 |
| Posterior parietal cortex | 3 -73 55 | 4.41 | p<.001 | 1020 |
| Cerebellum | 30 -55 -26 | 3.73 | p<.001 | 360 |
| Fusiform gyrus | -57 -43 -17 | 3.68 | p<.001 | 364 |
| Lateral frontopolar cortex | -27 53 4 | 3.53 | p=.013 | 196 |
