# Supplementary material for: The Neural Representation of Prospective Choice during Spatial Planning and Decisions
Source: PLoS Biol. 2017 Jan 12;15(1):e1002588. doi: 10.1371/journal.pbio.1002588 (PMC5231323; doi:10.1371/journal.pbio.1002588)
Supplement: S9 Table — List of peak voxels for clusters found in the Unchosen Path Length difference contrast. (DOCX) [file pbio.1002588.s016.docx]

**S9 Table**

| Region (Larger Path Differences) | MNI coordinates (xyz) | peak Z-score | Cluster corrected p-value | Cluster size (k) |
| --- | --- | --- | --- | --- |
| Angular gyrus | 51 -61 25 | 5.98 | p<.001 | 1138 |
| Posterior cingulate cortex | 12 -46 37 | 4.93 | p<.001 | 1200 |
| Ventral striatum | 27 8 1 | 4.58 | p=.005 | 206 |
| Region (Smaller Path Differences) | MNI coordinates (xyz) | peak Z-score | Cluster corrected p-value | Cluster size (k) |
| Lateral occipital complex | 27 -91 10 | 5.1 | p<.001 | 364 |
| Lateral occipital complex | -24 -88 4 | 4.71 | p<.001 | 549 |
| Dorsal anterior cingulate cortex | 9 17 34 | 4.41 | p=.008 | 192 |
