# Supplementary material for: The Neural Representation of Prospective Choice during Spatial Planning and Decisions
Source: PLoS Biol. 2017 Jan 12;15(1):e1002588. doi: 10.1371/journal.pbio.1002588 (PMC5231323; doi:10.1371/journal.pbio.1002588)
Supplement: S10 Table — List of peak voxels for clusters found in subsequent choice RT contrast. Please note that despite our stringent threshold (p < 0.005 activation threshold, cluster-based threshold p < 0.05), some activations are very large (k > 2,000) and span multiple brain regions. Consequently, the labels assigned to each cluster should be interpreted with caution. (DOCX) [file pbio.1002588.s017.docx]

**S10 Table**

| Region (Larger RT) | MNI coordinates (xyz) | peak Z-score | Cluster corrected p-value | Cluster size (k) |
| --- | --- | --- | --- | --- |
| Primary visual cortex | -12 -94 4 | 6.45 | p<.001 | 19102 |
| Middle temporal gyrus | 57 -25 -14 | 4.63 | p<.001 | 392 |
| Dorsal anterior cingulate cortex | 9 17 34 | 4.41 | p=.008 | 192 |
