# Supplementary material for: The Neural Representation of Prospective Choice during Spatial Planning and Decisions
Source: PLoS Biol. 2017 Jan 12;15(1):e1002588. doi: 10.1371/journal.pbio.1002588 (PMC5231323; doi:10.1371/journal.pbio.1002588)
Supplement: S11 Table — List of peak voxels for clusters found in the length of shortest available path contrast. Please note that despite our stringent threshold (p < 0.005 activation threshold, cluster-based threshold p < 0.05), some activations are very large (k > 2,000) and span multiple brain regions. Consequently, the labels assigned to each cluster should be interpreted with caution. (DOCX) [file pbio.1002588.s018.docx]

**S11 Table**

| Region (Distal) | MNI coordinates (xyz) | peak Z-score | Cluster corrected p-value | Cluster size (k) |
| --- | --- | --- | --- | --- |
| Inferior occipital cortex | -15 -97 -2 | 6.28 | p<.001 | 4431 |
| Dorsal anterior cingulate cortex | 12 23 34 | 4.33 | p=.011 | 242 |
| Region (Proximal) | MNI coordinates (xyz) | peak Z-score | Cluster-level FWE p-value | Cluster size (k) |
| Temporoparietal junction | -36 -52 28 | 4.93 | p<.001 | 1353 |
| Pregenual anterior cingulate cortex | -9 26 -17 | 4.55 | p<.001 | 477 |
| Rostrodorsal medial prefrontal cortex | -6 44 34 | 4.50 | p<.001 | 487 |
| Temporoparietal junction | 57 -64 28 | 4.49 | p=.001 | 360 |
| Precuneus | 0 -64 31 | 4.21 | p=.001 | 352 |
| Posterior superior temporal sulcus | 60 -37 1 | 3.94 | p<.001 | 385 |
| Lateral prefrontal cortex | -48 20 16 | 3.75 | p=.008 | 258 |
