# Supplementary material for: The Neural Representation of Prospective Choice during Spatial Planning and Decisions
Source: PLoS Biol. 2017 Jan 12;15(1):e1002588. doi: 10.1371/journal.pbio.1002588 (PMC5231323; doi:10.1371/journal.pbio.1002588)
Supplement: S12 Table — List of peak voxels for clusters found in the accuracy contrast. (DOCX) [file pbio.1002588.s019.docx]

**S12 Table**

| Region (Correct) | MNI coordinates (xyz) | peak Z-score | Cluster corrected p-value | Cluster size (k) |
| --- | --- | --- | --- | --- |
| Cerebellum | -18 -73 -38 | 4.11 | p<.001 | 421 |
| Cerebellum | 21 -79 -35 | 3.86 | p=.041 | 161 |
| Motor cortex | 0 -31 64 | 3.67 | p=.035 | 168 |
| Region (Incorrect) | MNI coordinates (xyz) | peak Z-score | Cluster corrected p-value | Cluster size (k) |
| Dorsal anterior cingulate cortex | 6 17 49 | 6.89 | p<.001 | 1290 |
| Anterior insula | 36 23 -5 | 6.19 | p<.001 | 1252 |
| Anterior insula | -30 23 -5 | 6.11 | p<.001 | 642 |
| Intraparietal sulcus | 51 -40 55 | 3.88 | p=.006 | 244 |
| Intraparietal sulcus | -45 -40 49 | 3.6 | p=.018 | 195 |
