# Supplementary material for: The Neural Representation of Prospective Choice during Spatial Planning and Decisions
Source: PLoS Biol. 2017 Jan 12;15(1):e1002588. doi: 10.1371/journal.pbio.1002588 (PMC5231323; doi:10.1371/journal.pbio.1002588)
Supplement: S13 Table — List of peak voxels for clusters found in contrasts for deep versus shallow interactions related to different parametric regressors. Please note that despite our stringent threshold (p < 0.005 activation threshold, cluster-based threshold p < 0.05), some of the activations are very large (k > 2,000) and span multiple brain regions. Consequently, the labels assigned to each cluster should be interpreted with caution. (DOCX) [file pbio.1002588.s020.docx]

**S13 Table**

| Region (Deep v Shallow Initial Path Differences) | MNI coordinates (xyz) | peak Z-score | Cluster corrected p-value | Cluster size (k) |
| --- | --- | --- | --- | --- |
| Posterior parietal cortex | -33 -82 28 | 5.40 | p<.001 | 2479 |
| Premotor cortex | -21 -4 64 | 4.93 | p<.001 | 782 |
| Dorsolateral prefrontal cortex | 48 35 34 | 4.52 | p=.007 | 243 |
| Premotor cortex | 27-4 61 | 4.47 | p=.012 | 218 |
| Region (Shallow v Deep RT) | MNI coordinates (xyz) | peak Z-score | Cluster corrected p-value | Cluster size (k) |
| Visual cortex | 9 -94 22 | 3.76 | p<.001 | 293 |
| Primary motor cortex | 42 -22 64 | 3.68 | p=.007 | 212 |
| Region (Deep V Shallow Length of Shortest Path) | MNI coordinates (xyz) | peak Z-score | Cluster corrected p-value | Cluster size (k) |
| Inferior temporal cortex | 48 -52 -14 | 5.33 | p<.001 | 2580 |
| Superior frontal gyrus | -24 23 37 | 4.29 | p<.001 | 1054 |
| Region (Deep V Shallow Accuracy) | MNI coordinates (xyz) | peak Z-score | Cluster corrected p-value | Cluster size (k) |
| Lateral occipital complex | -36 -76 19 | 4.31 | p<.001 | 533 |
| Posterior parietal cortex | -27 -64 64 | 4.25 | p<.001 | 456 |
| Intraparietal sulcus | 42 -34 43 | 4.23 | p<.001 | 1029 |
